# Motivated to care: latent classes of caregiver motivation as moderators among stress, resources, and well-being

**DOI:** 10.3389/fpsyg.2025.1608435

**Published:** 2025-11-12

**Authors:** Deborah L. Nichols, Amy Laine

**Affiliations:** 1 Department of Human Development and Family Science, Purdue University, West Lafayette, IN, United States; 2 SandwYch, LLC, Austin, TX, United States

**Keywords:** informal caregiving motivations, informal caregiving responsibilities, informal caregiver wellbeing, resilience, social support, informal caregiving integrative model (ICIM), latent class analysis, path modeling

## Abstract

**Introduction:**

Informal caregiving motivations are central to how informal caregivers interpret stressors, access resources, and maintain wellbeing. However, motivation is rarely integrated into models of caregiver stress. This study applies the Informal Caregiving Integrative Model to identify distinct motivation profiles and test their role in moderating the impact of caregiving responsibilities and resources on caregiver distress and burnout.

**Methods:**

Using data from 1,026 U.S. informal caregivers, latent class analysis identified five motivational profiles: Duty-, Affectively-, Obligation-, Culturally-, and Situationally-Motivated. Path models tested how these classes moderated the associations among caregiving tasks, duration, psychological resources (resilience, social support), and wellbeing outcomes (acute emotional distress, chronic burnout). Models were run in Stata using standardized observed variables and interaction terms.

**Results:**

Motivation profiles significantly moderated associations among caregiving responsibilities, resources, and wellbeing. Duty-Motivated informal caregivers experienced reduced burnout with more hours but increased strain under intensive direct care tasks. Affectively-Motivated informal caregivers showed vulnerability to distress from time and task demands but benefitted from resilience. Obligation-Motivated informal caregivers consistently reported high distress and burnout. Culturally-Motivated informal caregivers experienced mixed effects where cultural alignment buffered burnout under some conditions but not distress. Situationally-Motivated informal caregivers, while generally less anchored in enduring motives, provided the baseline against which other profiles’ stress–resource responses diverged. Resilience was broadly protective, while social support showed uneven and sometimes paradoxical associations.

**Discussion:**

Findings support informal caregiving motivation as a key contextual factor in informal caregivers’ stress response. Tailored interventions addressing motivational profiles may better support informal caregiver wellbeing than one-size-fits-all approaches.

## 1 Introduction

Over 53 million Americans provide unpaid care to family, friends, or loved ones (AARP and National Alliance for Caregiving, 2020). In this study, caregiving refers specifically to informal, unpaid care provided by a relative or friend. The tasks involved in informal caregiving are diverse and demanding, exerting their influence in varied ways. About 22% of caregivers report declines in physical health, and 36% experience increased stress (AARP and National Alliance for Caregiving, 2020). Prior research suggests that informal caregiving impacts vary depending on motivations, resources, and broader contexts, including social, cultural, and relational factors (Broxson and Feliciano, 2020). Although most caregiving studies originate in Western contexts, there is growing recognition that motivations are deeply shaped by national policies, cultural expectations, and historical caregiving traditions. For example, cross-national work from the EUROFAMCARE project indicated that duty was the most cited reason for caregiving in Greece and Poland, whereas emotional bonds predominated in Sweden (Huber et al., 2008). Meta-ethnographic syntheses have likewise shown that macro-level scripts like reciprocity, moral duty, spiritual fulfillment, and gendered expectations profoundly influence why people care and how they interpret that experience (Zarzycki and Morrison, 2021; Zarzycki et al., 2023). These influences operate alongside individual and family circumstances, suggesting that no single or universal pattern of caregiving motivation exists. Collectively, these findings emphasize the culturally embedded nature of caregiving motivations as opposed to a uniform orientation.

The Informal Caregiving Integrative Model (ICIM; Gérain and Zech, 2019) addresses this complexity by positing that caregiving motivations regulate reactions to stress and capacity to mobilize resources. These motivations develop from internal and external sources including societal norms, relational obligations, and pragmatic needs, and are viewed as dynamic rather than static. This dynamic perspective aligns with lifespan and ecological theories that emphasize how motivations may shift with changes in caregiver age, care recipient condition, or available supports. Unlike models that center solely on stressors and coping (e.g., Pearlin et al., 1990), the ICIM foregrounds cultural and relational contexts. It further positions motivation as a central mechanism linking context to caregiver outcomes, rather than a peripheral individual difference. This research examines three dimensions of caregiving: the formation of caregiving motivations through cultural norms and contextual pressures; the interaction between caregiving responsibilities and resources and how motivations moderate stress responses; and the influence of motivations on wellbeing outcomes.

### 1.1 Informal caregiving motivations and cultural contexts

Caregiving experiences vary in both intensity and meaning. While stress-process models emphasize objective burdens and role strain (Pearlin et al., 1990), recent research suggests that informal caregiving motivations significantly shape how informal caregivers appraise and respond to these challenges (Gérain and Zech, 2019; Quinn et al., 2010). Intrinsic motivations including love, identity alignment, or moral fulfillment may buffer against stress and enhance resilience. Conversely, extrinsic motivations develop when informal caregiving is driven by external pressures including social expectations, financial necessity, or a lack of viable alternatives (Adelman et al., 2014; Deci and Ryan, 2008; Quinn and Toms, 2019). Importantly, intrinsic and extrinsic motives are not mutually exclusive; informal caregivers often navigate complex blends that may generate both protective and risk processes simultaneously. In practice, informal caregivers often endorse a blend of intrinsic and extrinsic motivations that likely vary in salience and shift depending on relational dynamics, care demands, and contextual pressures (Barry et al., 2021; Zarzycki and Morrison, 2021; Zarzycki et al., 2023).

Prior research rooted in Self-Determination Theory (SDT) has provided a robust theoretical framework for conceptualizing motivational differences, emphasizing a continuum from externally regulated to fully internalized informal caregiving motives (Deci and Ryan, 2008). Possessing more internalized and autonomous motives alongside greater identification with the informal caregiving role predicted more positive informal caregiver outcomes while externally driven motives predicted higher burden and more depressive symptoms (Barry et al., 2021). Similarly, informal caregivers reporting high levels of autonomy, competence, and relatedness in their role as caregivers also reported higher levels of intrinsic motivation and subsequently more positive wellbeing (Dombestein et al., 2020). However, most SDT-based studies have not incorporated cultural variability in motivational structure, potentially limiting the applicability of their findings across diverse informal caregiving populations.

While this SDT-focused research provides evidence that the nature of informal caregiving motivations is linked to its psychological consequences, this research typically adopts a variable-centered approach that treats motives as operating along a singular continuum. In contrast, the current study applies a person-centered approach using latent class analysis (LCA) to construct discrete motivation profiles or typological constellations of co-occurring informal caregiving motives. This distinction is critical. Rather than treating motivations as additive or linear, LCA captures how real-world informal caregivers may hold overlapping and sometimes conflicting reasons for informal care that interact in patterned ways. It also enables the analysis of how these profiles moderate the effects of informal caregiving stressors and resources on wellbeing.

The EUROFAMCARE Consortium (2006) project (Huber et al., 2008) provided a foundational framework for this approach. Based on a factor analysis of 10 common informal caregiving reasons across six European countries, EUROFAMCARE identified five motivational domains: duty, external determination, emotional ties, religious beliefs, and financial necessity. These categories informed the current study’s item selection. Cross-national analyses in EUROFAMCARE revealed that motivational emphasis varied by both country and care context. For example, duty was strongest in Greece and Poland, while emotional bonds predominated in Sweden. Such patterns highlight the need for empirical approaches that can capture the multidimensionality of motives while respecting their cultural specificity. While EUROFAMCARE examined shared variance among motives using factor analysis, the present study builds on that work by using LCA to identify distinct informal caregiver motivation types and to test how those profiles function as moderators in stress and support pathways.

This study is also informed by more recent qualitative and meta-ethnographic work emphasizing sociocultural context. Zarzycki et al. (2022, 2023) synthesized findings across informal caregiving cultures to reveal how macro-level scripts like reciprocity, moral duty, spiritual fulfillment, and gendered expectations influence informal caregiver identity and strain. This research is confirmed in qualitative studies from Latin, Asian, and European contexts indicating that cultural norms influence both why people care and how they interpret the experience (Knight and Sayegh, 2010; Cook et al., 2018). In combination, these lines of evidence suggest that typological profiles rooted in both psychological theory and cross-cultural research may provide a more ecologically valid basis for predicting informal caregiver outcomes. Despite this growing literature, most prior research has examined individual motives or motivational quality in isolation. Few studies have empirically identified clusters of informal caregivers who endorse similar combinations of motives and even fewer have tested how these profiles interact with stressors and resources to influence psychological outcomes. The present study directly addresses this gap by linking motivational profiles to wellbeing through a multivariate, contextualized lens.

In the U.S. context, racial and ethnic identity often functions as a proxy indicator of cultural norms and informal caregiving scripts, reflecting differences in expectations, values, and support structures (Knight and Sayegh, 2010; Dilworth-Anderson and Gibson, 2002). Because a core aim of this study is to examine motivational profiles within their broader sociocultural contexts, race and ethnicity were included alongside motivational indicators in the latent class model. This approach is consistent with prior informal caregiving research that treats race and ethnicity as culturally embedded variables, enabling identification of profiles that reflect both psychological motives and the cultural contexts in which those motives are enacted (Dilworth-Anderson et al., 2002; Pinquart and Sörensen, 2005).

### 1.2 Informal caregiving stressors and available resources

The ICIM defines informal caregiving stressors as central determinants of informal caregiver strain and wellbeing. The ICIM extends the Stress Process Model (Pearlin et al., 1990) by explicitly incorporating informal caregiving motivations as dynamic moderators of the stress–outcome relationship, and it integrates principles from SDT (Deci and Ryan, 2008) within an ecological framework. This study focuses specifically on primary stressors, which are objective, quantifiable indicators of informal caregiving burden. These include informal caregiving duration, weekly informal caregiving hours, and task-related burden including the provision of intensive personal care and the coordination or management of care (Pearlin et al., 1990). These indicators represent the structural and time-based demands placed on informal caregivers, independent of their subjective appraisals.

The impact of primary stressors on wellbeing is not uniform. The ICIM emphasizes that outcomes depend on the availability and quality of personal resources, external supports, and cultural assets. Internal psychological resources like resilience, operationalized here as a combination of self-esteem, mastery, and optimism (Julian et al., 2022), equip informal caregivers to manage stress and preserve wellbeing. External resources, including social support and financial stability, alleviate practical informal caregiving demands and can bolster coping capacity (Padmanabhanunni et al., 2023). Cultural assets, including prevailing norms around duty, reciprocity, or family care, further influence how informal caregiving is perceived and supported. Because informal caregiving in the United States occurs within a culturally diverse landscape, including intersecting differences by ethnicity, gender, and immigration status, it is essential to validate whether these motivational profiles and their moderating effects hold across such varied contexts. These assets can enhance resource availability or undermine it, depending on alignment with informal caregivers’ motivational orientation (Schulz and Sherwood, 2008).

Critically, the protective power of any resource is moderated by the degree to which informal caregiving is experienced as voluntary, meaningful, and identity-consistent. Informal caregivers possessing more intrinsic motivations may reframe stressors as worthwhile and, consequently, be more effective at mobilizing support and using adaptive coping strategies. In contrast, informal caregivers with more extrinsic motivations may feel coerced, financially trapped, or unsupported and, as a result, struggle to benefit from resources, perceive themselves to have less autonomy, and report higher psychological distress. Other research indicates that this alignment between motivation, sociocultural values, and support systems is essential to understanding both the sustainability and strain of informal caregiving (Sharma et al., 2016; Zarzycki et al., 2022, 2023).

### 1.3 Informal caregiver wellbeing: acute distress and chronic burnout

The ICIM conceptualizes informal caregiver wellbeing as continually changing and capable of producing immediate and cumulative effects (Gérain and Zech, 2019). Acute emotional distress represents informal caregivers’ short-term psychological responses to the immediate unpredictability and intensity of informal caregiving demands, often manifested as symptoms of anxiety and depression (Kroenke et al., 2003, 2007). When informal caregiving responsibilities conflict with motivations, distress is heightened, especially when personal resilience or social support is lacking. Conversely, when motivations and informal caregiving roles align with one’s self-concept and values, informal caregivers are better able to reframe stressors as meaningful, buffering against distress. Acute distress is a proximal indicator of informal caregiving strain and may serve as an early warning sign of impending burnout (Gérain and Zech, 2019; Kindt et al., 2015). While resilience and social support buffer distress, these protective effects are contingent on alignment between motivations and responsibilities. Because acute distress is typically more responsive to immediate relief including crisis intervention, respite care, or short-term emotional support, its early identification can help prevent progression to chronic strain.

Chronic burnout, defined by emotional exhaustion and depersonalization (Trockel et al., 2017), represents the cumulative impact of prolonged exposure to informal caregiving demands. Unlike acute distress, burnout develops gradually as informal caregiving responsibilities deplete personal and social resources over time. Persistent stressors like financial strain, role conflicts, or informal caregiving in isolation without adequate support accelerate this depletion. Within the ICIM, burnout functions as both an outcome of informal caregiving strain and a mediator linking informal caregiving demands to broader mental and physical health outcomes. It results from the culmination of stress, particularly when informal caregivers feel they have diminishing control over their roles and resources. For intrinsically-motivated informal caregivers, burnout is likely to occur when overcommitment erodes their internal sense of self, especially in the absence of adequate self-care. Conversely, extrinsically-motivated informal caregivers are more prone to burnout when expectations are unmet, autonomy is lacking, and available resources are insufficient (Kindt et al., 2015). Addressing burnout, which is rooted in sustained role overload, often requires long-term structural supports like workload redistribution, sustained respite, and ongoing peer or professional support rather than solely acute interventions.

Because informal caregiver wellbeing is closely linked to the quality of life, social participation, and emotional health of both the informal caregiver and care recipient, particularly in the context of aging, it is critical to understand how motivational factors influence the sustainability of informal care. Informal caregiver health has been identified as a major determinant of care recipient outcomes, including risk of institutionalization (Reinhard et al., 2008). Informal caregiving often leads to declines in mental and physical quality of life over time (Keramat et al., 2025). Conversely, interventions that support informal caregivers like counseling, education, and respite care can directly reduce informal caregiver distress and, indirectly, improve care recipient wellbeing (Reinhard et al., 2008; Sanchís-Soler et al., 2025). Supporting informal caregiver wellbeing ultimately promotes healthier aging trajectories and better quality-of-life outcomes for older adults.

### 1.4 Research gap and study purpose

Despite a growing literature on informal caregiving motives, most prior research has examined individual motives or motivational quality in isolation, often within single cultural or demographic groups, and primarily through variable-centered approaches. This gap is important because most existing informal caregiver research uses variable-centered methods that average across individuals, obscuring the heterogeneous subgroups that are critical for developing clinically relevant, targeted supports. Few studies have empirically identified typological profiles of informal caregivers that capture overlapping or conflicting motives, and even fewer have tested how these profiles interact with caregiving stressors and resources to influence both acute and chronic wellbeing outcomes.

The present study directly addresses this gap by integrating a person-centered analytic approach with a culturally informed theoretical framework to examine the moderating role of motivation profiles in stress and support pathways. Unlike variable-centered models, LCA empirically identifies patterns of co-occurring motives that may not be apparent when examining each motive in isolation, providing more ecologically valid profiles that can guide tailored interventions. Drawing on the ICIM, this study uses a person-centered LCA to identify distinct motivational profiles that reflect real-world constellations of informal caregiving motives. It then tests how these profiles moderate the associations between informal caregiving stressors, internal and external resources, and two key wellbeing outcomes: acute emotional distress and chronic burnout. This study addresses three key objectives: (1) to identify latent classes of informal caregiving motivation based on responses to multiple informal caregiving motive indicators; (2) to examine demographic and psychological correlates that predict membership in each motivation class; and (3) to test whether informal caregiving motivation profiles moderate the effects of informal caregiving stressors and resources on informal caregiver wellbeing, as reflected in both acute and chronic psychological strain.

### 1.5 Hypotheses

Based on the ICIM and prior empirical work, the following hypotheses are proposed: (1) informal caregivers will cluster into distinct latent classes characterized by different constellations of intrinsic and extrinsic motives; (2) the protective effects of resilience and social support on informal caregiver wellbeing will be stronger among intrinsically motivated informal caregivers than among extrinsically motivated informal caregivers; and (3) the strength and direction of associations between informal caregiving stressors, resources, and wellbeing will differ by motivation class, with intrinsic-dominant classes showing greater benefit from resilience and social support, and extrinsic-dominant classes showing weaker or null protective effects. By identifying distinct motivational profiles and testing their moderating role in stress and support pathways, this study contributes both to theoretical refinement of the ICIM and to the practical development of culturally responsive, motivation-targeted informal caregiver interventions.

## 2 Method

### 2.1 Participants

#### 2.1.1 Informal caregivers

The sample consisted of 1,026 informal caregivers aged 18 to 80 years (*M* = 38.61, *SD* = 12.48) who provided unpaid care within the previous 12 months. The majority were women (66.0%), with 32.7% identifying as men, and 1.3% as non-binary or another gender identity. Informal caregivers identified as 54.8% White, 17.6% Black or African American, 7.2% Asian American, 2.2% American Indian or Alaska Native (AIAN), and 3.1% Other, with 15.4% reporting their ethnicity as Hispanic or Latino. Most informal caregivers were partnered (49.4%), with 37.2% single, 9.1% divorced, and 4.3% widowed. More than 43% reported leaving their jobs to provide care, while 35.5% were currently employed, and 20.7% were not employed. Educational attainment varied, with 53.3% holding undergraduate or postgraduate degrees, 27.8% having completed some college, 13.3% holding a high school diploma, 3.9% receiving vocational or trade school training, and fewer than 2% not completing high school. About 52% reported an annual income of $59,000 or less, while 15% reported earning $100,000 or more. Data collection occurred between August 2022 and August 2023 across the United States, with participants from the Northeast (18.9%), Midwest (21.1%), South (38.0%), and West (22.0%). See Table 1 for additional details.

**Table 1:** Demographics, informal caregiving responsibilities, and available resources overall and by informal caregiving motivation class.

Category | Variable	Overall	C1	C2	C3	C4	C5	Χ^2^ |*t*-test
Informal caregiver demographics							
Gender (women)	67.3%	53.3%^a^	70.4%^b^	73.3%^b^	68.0%^ab^	62.1%^ab^	15.63**
AIAN	2.2%	3.3%^a^	1.5%^a^	3.1%^a^	3.3%^a^	1.7%^a^	2.96
Asian	7.2%	15.6%^a+^	6.4%^b^	1.0%^c-^	19.7%^a+^	3.8%^bc-^	52.87***
Black	17.6%	15.6%^a^	21.4%^a^	12.0%^a^	19.7%^a^	15.7%^a^	9.08
White	54.8%	40.0%^ab-^	57.7%^c^	72.8%^d+^	27.9%^b-^	54.9%^ac^	69.94***
Other	3.1%	5.6%^a^	2.1%^a^	2.6%^a^	2.5%^a^	4.7%^a^	5.44
of Hispanic or Latino Ethnicity	15.4%	20.0%^ab^	11.3%^b-^	8.9%^b-^	27.0%^a+^	19.6%^a^	28.42***
Age	38.61 (0.39)	41.33^a^ (1.30)	38.05^bcd^ (0.63)	41.53^a^ (0.90)	36.15^ce^ (1.12)	37.38^de^ (0.81)	5.74***
Education | Employment							
Education Level^1^	3.18 (0.05)	3.59^a^ (0.15)	3.09^bc^ (0.07)	3.06^bd^ (0.10)	3.84^a^ (0.13)	2.93^cd^ (0.09)	10.53***
Not employed	20.7%	12.2%^a^	24.0%^a^	18.3%^a^	13.1%^a^	24.3%^a^	13.23**
Was employed, but stopped	43.9%	48.9%^a^	41.8%^a^	46.6%^a^	47.5%^a^	41.3%^a^	3.51
Currently employed	35.5%	38.9%^a^	34.3%^a^	35.1%^a^	39.3%^a^	34.5%^a^	1.62
Income^2^	6.00 (0.12)	6.34^ac^ (0.40)	5.89^ab^ (0.19)	6.35^ac^ (0.27)	6.74^c^ (0.34)	5.38^b^ (0.25)	3.41**
Relationship status							
Single	37.2%	36.7%^a^	37.1%^a^	29.8%^a^	39.3%^a^	42.6%^a^	7.56
Married/Partnered	49.7%	42.2%^ab^	50.0%^ab^	57.6%^b^	53.3%^ab^	43.8%^a^	10.65*
Divorced/Separated	8.9%	12.2%^a^	8.2%^a^	8.9%^a^	4.9%^a^	10.6%^a^	4.70
Widowed	4.7%	7.8%^a^	5.2%^a^	4.7%^a^	3.3%^a^	3.4%^a^	3.53
Living situation							
Alone	19.4%	32.2%^a+^	18.0%^b^	19.4%^ab^	16.4%^ab^	18.3%^ab^	10.81*
With Spouse/Partner	59.2%	51.1%^a^	60.8%^a^	60.2%^a^	60.7%^a^	57.9%^a^	3.22
With parents	8.0%	5.6%^a^	9.0%^a^	6.8%^a^	9.0%^a^	7.7%^a^	1.86
With roommates/ Other	13.5%	11.1%^a^	12.1%^a^	13.6%^a^	13.9%^a^	16.2%^a^	2.54
Care recipient demographics							
Gender (women)	60.4%	57.8%^a^	62.4%^a^	59.2%^a^	68.0%^a^	55.3%^a^	6.52
AIAN	1.7%	4.4%^a+^	1.5%^a^	2.1%^a^	1.6%^a^	0.4%^a^	6.73
Asian	6.7%	15.6%^a+^	5.4%^b^	1.0%^b-^	19.7%^a+^	3.4%^b-^	58.80***
Black	17.3%	14.4%^a^	20.4%^a^	13.6%^a^	18.9%^a^	15.3%^a^	5.73
White	60.2%	46.7%^ab^	63.1%^c^	76.4%^d+^	34.4%^b-^	60.9%^ac^	63.19***
Other	3.9%	7.8%^a+^	2.3%^a^	2.6%^a^	6.6%^a^	4.7%^a^	9.72*
Of Hispanic or Latino Ethnicity	15.7%	18.9%^abcd^	12.1%^cd^	12.0%^bd^	25.4%^a+^	18.3%^abcd^	16.29**
Has a Spouse	33.2%	28.9%^a^	36.3%^a^	31.9%^a^	34.4%^a^	30.2%^a^	3.64

Category | Variable	Overall	C1	C2	C3	C4	C5	Χ^2^|*t*-test
Age	61.72 (0.65)	67.41^a^ (2.20)	62.20^b^ (1.06)	60.75^bc^ (1.51)	64.89^ab^ (1.89)	57.90^c^ (1.36)	4.50**
Living situation							
In Own Home	74.1%	70.0%^a^	76.0%^a^	66.0%^a^	77.0%^a^	77.4%^a^	10.04*
In Someone Else’s Home	9.0%	12.2%^a^	7.2%^a^	8.9%^a^	13.9%^a^	8.1%^a^	6.54
In a Facility	5.6%	5.6%^a^	7.2%^a^	4.7%^a^	4.1%^a^	4.6%^a^	3.55
With Primary Caregiver	8.9%	8.9%^abc^	7.7%^c^	15.7%^b+^	4.1%^ac^	7.7%^abc^	15.53
Care recipient is caregiver’s…							
Spouse	9.9%	10.0%^abcd^	11.3%^cd^	15.2%^bd+^	1.6%^a-^	7.7%^abcd^	17.45***
Parent (in-law, step)	42.3%	55.6%^a^	40.1%^ab^	44.5%^ab^	46.7%^ab^	36.6%^b^	11.74*
Grandparent (in-law)	16.4%	16.7%^a^	19.5%^a^	12.0%^a^	21.3%^a^	11.9%^a^	11.06*
Child, Grandchild, Niece, Nephew	5.3%	2.2%^a^	4.4%^a^	9.9%^a+^	2.5%^a^	5.5%^a^	12.67*
Older Relative (aunt, uncle, godparent)	6.7%	2.2%^a^	8.0%^a^	4.7%^a^	9.0%^a^	6.8%^a^	6.13
Sibling, Cousin	5.6%	6.7%^a^	4.9%^a^	7.3%^a^	6.6%^a^	4.7%^a^	2.22
Friend, Neighbor, Roommate, Colleague	13.8%	6.7%^a^	11.8%^a^	6.3%^a-^	12.3%^a^	26.8%^b+^	47.78***
Caregiving responsibilities							
Caregiving hours per week	32.31 (1.03)	41.11^a^ (3.48)	31.55^bc^ (1.67)	35.31^acd^ (2.39)	30.01^bd^ (2.99)	28.94^b^ (2.15)	2.81*
Years of care	4.19 (0.17)	5.27^a^ (0.56)	3.71^b^ (0.27)	5.15^c^ (0.38)	4.60^abc^ (0.48)	3.57^b^ (0.35)	4.28**
Intensive personal care	3.93 (0.08)	5.23^a^ (0.29)	3.81^b^ (0.14)	4.53^c^ (0.20)	3.36^b^ (0.25)	3.45^b^ (0.18)	9.99***
Care coordination	4.04 (0.06)	5.57^a^ (0.20)	3.75^bc^ (0.10)	4.70^d^ (0.14)	3.90^c^ (0.18)	3.46^b^ (0.13)	27.26***
Care recipient’s health challenges							
Memory problems	21.6%	35.6%^a+^	19.6%^bc^	28.8%^ac+^	20.5%^abc^	14.5%^b-^	24.23***
Short, term physical condition (e.g., surgery)	21.5%	22.2%^a^	22.4%^a^	19.4%^a^	18.0%^a^	23.4%^a^	2.11
Long, term physical condition (e.g., ALS, Alzheimer’s)	28.9%	45.6%^a+^	28.9%^b^	36.1%^ab^	25.4%^bc^	18.7%^c-^	29.54***
Chronic condition (e.g., diabetes, heart disease)	36.3%	48.9%^a+^	34.8%^ab^	45.0%^a+^	34.4%^ab^	27.7%^b-^	20.62***
Emotional or mental health issue	29.9%	31.1%^abc^	27.6%^c^	39.8%^b+^	29.5%^abc^	25.5%^ac^	12.12*
Behavioral Problems	8.3%	8.9%^a^	6.2%^a^	11.0%^a^	12.3%^a^	7.2%^a^	7.06
Developmental or intellectual disorder	4.3%	1.1%^a^	3.9%^a^	7.3%^a+^	4.1%^a^	3.8%^a^	6.82
General concerns to aging	38.8%	48.9%^a^	38.9%^ab^	42.9%^aab+^	40.2%^ab^	30.6%^b-^	11.92*
Total problems (Mean)	1.90 (0.04)	2.42^a^ (0.12)	1.82^bc^ (0.06)	2.30^a^ (0.08)	1.84^c^ (0.10)	1.51^d^ (0.07)	17.71***

*p < 0.05; **p < 0.01; **p < 0.001; When superscript letters in a particular row differ, the column proportions/means for those two comparisons differ significantly at the *p* < 0.05 level. (+) The standardized residual was greater than 1.96 indicating that total cases in this cell were significantly greater (*p* < 0.05) than expected; (−) The standardized residual was less than −1.96 indicating that total cases in this cell were significantly fewer (*p* < 0.05) than expected. ^1^Informal caregiver education: 0 = < High School (HS), 1 = HS/GED, 2 = Some College, 3 = Vocational/Trade School, 4 = Undergraduate degree, 5 = Postgraduate degree; ^2^Informal caregiver income: 1 = <$20,000, categories 2–9 increase by $10,000, 10 = $100,000–$124,000, categories 11–13 increase by $25,000, 14= > $200,000.

#### 2.1.2 Care recipients

Care Recipients ranged in age from 2 months to 102 years (*M* = 61.72, *SD* = 21.00), with 75.2% aged 50 years or older. Among those receiving care, 59.1% identified as women and 39.6% as men. In terms of racial identity, 60.2% identified as White, 17.3% as Black or African American, 6.7% as Asian, 1.7% as AIAN, and 6.7% as another racial identity. Additionally, 15.7% reported Hispanic or Latino ethnicity. Regarding living arrangements, 76.8% lived in their own homes, 13.5% resided in someone else’s home, 8.9% lived with their primary informal caregiver, and 5.7% were in a care facility. More than 33% of individuals receiving care reported having a spouse.

### 2.2 Sampling strategy

Participants were recruited through User Interviews, an online research recruitment platform that maintains a proprietary panel of about six million registered individuals. The platform enables researchers to target specific populations, including both consumer and professional segments, using screening questionnaires to determine eligibility. For this study, potential participants were identified through the platform’s screening process and invited to complete an online survey. The panel is monitored for data quality and participant authenticity through both automated and manual review processes, including profile verification, screening response checks, and activity monitoring. These processes are designed to minimize fraudulent participation and misrepresentation. Only individuals who met all eligibility criteria and provided informed consent were included in the sample.

Stratified quota sampling was implemented to balance participants across U.S. regions (Northeast, Midwest, South, West), race and ethnicity, and income levels. Screening confirmed that respondents had provided unpaid care to a relative or friend within the prior 12 months, with verification of informal caregiving role, duration, and recipient status. Invitations were sent with a three-day response window; non-responders were replaced sequentially with eligible participants to meet quotas. To reduce self-selection bias, participants received a nominal stiped of $10 dollars for completing the survey, administered directly by the recruitment firm. Of the 5,221 respondents who completed the initial screening, 3,458 met eligibility criteria. Ultimately, 1,062 eligible informal caregivers were invited to participate, and 1,026 provided complete responses.

#### 2.2.1 *A priori* sample size calculation

An *a priori* power analysis was conducted using G*Power (version 3.1.9.7; Faul et al., 2009) to estimate sample size. The analysis assumed a significance level of *α* = 0.05, power (1 − *β*) of 0.80, and a model with 31 predictors and 28 interaction terms. Effect sizes (𝑓2) were evaluated over a range of values from 0.02 (small) to 0.15 (medium). Based on this range, the estimated total sample size requirements ranged from 1,267 for 𝑓2 = 0.02 to 200 for 𝑓2 = 0.15. The available sample size of 1,026 exceeds the requirements for detecting effects as small as 𝑓2 = 0.025. Prior research supports moderately small to medium effect sizes for stressors, resources, and wellbeing in informal caregiver populations (del-Pino-Casado et al., 2021; Gilsenan et al., 2022; Suhr et al., 2020). The study is adequately powered to detect effects consistent with the literature while accommodating the complexity of the model. To clarify scope, the count of 31 predictors and 28 interaction terms reflects the full set of main effects (informal caregiving stressors, resources, class dummies, and demographics) and all prespecified two-way class-by-stressor/resource interactions tested in the moderation models, consistent with the study aims. Effect-size assumptions were anchored to meta-analytic estimates for informal caregiver stress and psychological outcomes, justifying the small-to-moderate range.

### 2.3 Data collection procedures

Data were collected between August 2022 and August 2023 via a secure web-based survey platform accessible on desktop, tablet, or mobile devices. The survey required approximately 25 min to complete. No personally identifying information was stored with survey responses. Each participant received a unique survey link to prevent duplicate entries. Upon completion, participants received compensation through the recruitment firm. Institutional Review Board approval was obtained prior to data collection (Purdue University Protocol ID: IRB-2022-827).

### 2.4 Measures

All measures were drawn from instruments with established psychometric properties in informal caregiver or general adult populations. Selection prioritized reliability, validity, and relevance to the study constructs while minimizing participant burden. Abbreviated subscales were used when prior research demonstrated strong psychometric performance, and all instruments were administered in their validated formats unless noted. Internal consistency in the present sample ranged from Cronbach’s *α* = 0.78–0.94 for multi-item scales and KR-20 = 0.66–0.78 for dichotomous informal caregiving task scales. Full reliability, validity, and scoring details are provided below where relevant.

#### 2.4.1 Demographics

Informal caregivers reported their own and their care recipient’s gender, age, living arrangements, spousal status, and zip code, along with employment, education, income, family composition, and relationship status. They also indicated whether care recipients experienced any of eight health conditions, summed to create a total challenges score (*M* = 1.90, SD = 1.18). Demographic indicators followed U.S. Census Bureau (Office of Management and Budget [OMB], 2024) standards. See Table 1.

#### 2.4.2 Informal caregiving motivations and cultural contexts

##### 2.4.2.1 Informal caregiving motivations

Eleven binary (yes/no) items from the EUROFAMCARE survey (2006) assessed informal caregiving motives. For transparency, items are grouped into four conceptual dimensions from the original EUROFAMCARE factor analysis: emotional bonds, duty, external constraint, and obligation (Table 2). Participants marked yes if the statement described one of their reasons for providing informal care (coded 1 = yes, 0 = no). These 11 indicators served as observed variables in the latent class analysis (LCA).

**Table 2:** Informal caregiving motivation items grouped by conceptual dimension (EUROFAMCARE indicators).

Dimension	Items
Duty | Obligation	An informal caregiver’s personal sense of obligation toward the person needing care as a family member An informal caregiver’s sense of duty
Emotional Bonds	Emotional bonds between informal caregiver and person needing care (love, affection) Caring for loved one makes informal caregiver feel good Person receiving care would not wish for anyone else to care for them
Cultural | Religious Beliefs	Because of informal caregiver’s cultural beliefs Because of informal caregiver’s religious beliefs
Constraint | External Determination	Cost of professional care would be too high There were economic benefits for informal caregiver or person needing care Informal caregiver found themselves in these circumstances almost by chance without making a decision Informal caregiver had no alternative

##### 2.4.2.2 Racial and ethnic identity

Informal caregivers reported race [American Indian or Alaska Native (AIAN), Asian, Black or African American, White, or other] and whether they identified as Hispanic or Latino, following U.S. Census Bureau (Office of Management and Budget, 2024) categories.

#### 2.4.3 Informal caregiving stressors

Informal caregiving stressors included weekly informal caregiving hours, total years of informal caregiving, and completion of 18 informal caregiving tasks. Tasks spanned activities of daily living (ADLs; Katz et al., 1963), instrumental activities of daily living (IADLs; Lawton and Brody, 1969), and medical/nursing tasks (AARP and National Alliance for Caregiving, 2020). Example items include “help with bathing or showering” (ADL) and “managing finances” (IADL). Factor analysis identified two categories: Intensive Personal Care (KR–20 = 0.78) and Care Coordination (KR–20 = 0.66). These categories are consistent with prior research demonstrating that ADLs, IADLs, and medical/nursing tasks form reliable, conceptually distinct domains and predict informal caregiver outcomes such as burden, stress, and time demands (Katz et al., 1963; Lawton and Brody, 1969; Schulz and Eden, 2016; Wolff et al., 2018). Detailed descriptions of these informal caregiving tasks and factor analysis results are provided in Supplementary Tables S1–S4.

#### 2.4.4 Psychological and social resources

Psychological and social resources were assessed using the Resilience Resources Scale (RRS; Julian et al., 2022), a brief multidimensional measure designed to capture protective factors across domains of self-esteem, mastery, optimism, social support, and familism. Six items from the self-esteem, mastery, and optimism subscales were combined to create an overall resilience composite (Cronbach’s *α* = 0.94 in this sample). These items were also grouped into three two-item subscales: Self-Esteem (α = 0.87), Mastery (α = 0.87), and Optimism (α = 0.86). Three additional RRS items formed a Social Support composite (α = 0.87), with a two-item Social Support–Seeking subscale (α = 0.81), and a single-item measure of familism (*I feel a strong responsibility to care for my family members regardless of personal cost*). Example items include: *I feel confident in my ability to handle difficult situations* (self-esteem), *I can solve most problems if I try hard enough* (mastery), *I generally expect good things to happen in my life* (optimism), *I can count on my friends when things go wrong* (social support), and the familism item above.

The RRS demonstrates strong reliability and validity across multiple samples, including undergraduate, community adult, and informal caregiving populations. Julian et al. (2022) reported internal consistency coefficients ranging from 0.81 to 0.94 across subscales, and confirmatory factor analyses supported the hypothesized five-factor structure. Convergent validity was evidenced through positive associations with established resilience measures (e.g., Connor-Davidson Resilience Scale, Brief Resilience Scale) and related constructs including life satisfaction and wellbeing, while discriminant validity was indicated by weaker correlations with unrelated constructs including social desirability. Criterion validity was established via significant negative associations with depressive symptoms, anxiety, and perceived stress, and positive associations with coping and social functioning. These findings justify the use of abbreviated subscales when item-level reliability is maintained, as in the present study. Standardized coefficients for these resources split across motivation classes are provided in Supplementary Table S5.

#### 2.4.5 Informal caregiver wellbeing

##### 2.4.5.1 Acute emotional distress

Acute emotional distress was assessed using a composite of the Generalized Anxiety Disorder–2 (GAD-2; Kroenke et al., 2007) and the Patient Health Questionnaire–2 (PHQ-2; Kroenke et al., 2003, 2007), which together showed strong internal consistency in this sample (Cronbach’s *α* = 0.89). These brief, validated measures capture core symptoms of anxiety and depression, two highly comorbid affective states that commonly co-occur in informal caregivers under acute strain; combining them provides a more comprehensive index of immediate emotional burden. The PHQ-2 demonstrates strong criterion validity for detecting major depressive disorder in primary care populations, with sensitivity of 82.9% and specificity of 90.0% at a cut-off score of 3. Construct validity is supported by high correlations with the PHQ-9 and clinician-administered diagnostic interviews. It is widely used as an efficient first-stage depression screener in diverse adult populations, including those with chronic illness and informal caregiving responsibilities. The GAD-2 similarly shows strong criterion validity for generalized anxiety disorder, with sensitivity of 86% and specificity of 83% at a cut-off score of 3. Convergent validity is evidenced by substantial correlations with the GAD-7 and with other anxiety disorder diagnoses, including panic disorder, social anxiety disorder, and PTSD. It has been validated as a brief and accurate screener across primary care and general adult samples, including populations with comorbid medical and psychological conditions.

##### 2.4.5.2 Chronic burnout

Chronic burnout was assessed with eight items adapted from the Stanford Professional Fulfillment Index (Trockel et al., 2017), evaluating emotional exhaustion and depersonalization. Participants reported symptom frequency over the past month on a 4-point scale (0 = never to 3 = nearly every day). Internal consistency in the present sample was high (Cronbach’s α = 0.89). The burnout subscales of the PFI (emotional exhaustion and depersonalization) have demonstrated strong construct validity through confirmatory factor analyses and significant associations with related constructs such as work overload, depressive symptoms, and turnover intentions. Criterion validity is supported by their ability to distinguish between groups differing in burnout risk (e.g., by hours worked or specialty) and by strong correlations with the Maslach Burnout Inventory emotional exhaustion and depersonalization subscales. The measure has been validated in physicians and other healthcare workers, with emerging use in informal caregiver populations.

### 2.5 Data analysis

#### 2.5.1 Purpose 1: identify distinct informal caregiving motivation classes

##### 2.5.1.1 Latent class analysis (LCA)

A LCA using MPlus v.8.8 (Muthén and Muthén, 2000) was conducted to identify naturally occurring subgroups of informal caregivers based on their motivational patterns, race, and ethnicity. This person-centered approach was selected to detect latent heterogeneity in the population that may not be observable through variable-centered methods, thereby capturing the complex combinations of motives that define distinct informal caregiving profiles (Masyn, 2013; Nylund-Gibson and Choi, 2018). Models specifying one to six classes were evaluated using multiple fit indices: Bayesian Information Criterion (BIC), Sample-size Adjusted BIC, Consistent Akaike Information Criterion, Approximate Weight of Evidence Criterion, and entropy values (Masyn, 2013; Nylund-Gibson and Choi, 2018; see Table 3). Race and ethnicity were entered into the LCA alongside motivational indicators to allow identification of profiles that jointly reflect psychological motives and culturally patterned informal caregiving contexts. This modeling choice was consistent with the study’s aim to examine informal caregiving meaning within the ICIM framework, where sociocultural background is a critical context for interpreting motivations. While race and ethnicity are not direct measures of cultural beliefs or practices, they serve as a pragmatic proxy for cultural norms in U.S.-based informal caregiving research, as documented in prior work (Dilworth-Anderson and Gibson, 2002; Knight and Sayegh, 2010).

**Table 3:** Latent class analysis fit indices and classification coefficients.

*K*	*d*	LL	BIC	SABIC	CAIC	AWE	BLRT *p*	VLMR-LRT *p*	Entropy	*BF*	*cmP*	%large	%small
1	13.00	−7213.63	14517.39	14476.10	14479.40	14485.90	--	--	--	0.00	0.00		
2	27.00	−7019.97	14227.13	14141.38	14148.24	14161.74	0.0001	**0.0001**	0.56	0.11	0.02	63.5%	36.5%
3	41.00	−6949.67	14183.62	14053.4	14063.80	14084.30	0.0001	0.0491	0.58	0.29	0.17	47.3%	18.9%
4	55.00	−6888.63	**14158.59**	13983.91	13997.87	14025.37	0.0001	0.4734	0.65	2.55	**0.58**	36.0%	13.7%
5	69.00	−6849.44	14177.28	**13958.13**	**13975.65**	**14010.15**	0.0001	0.0321	**0.70**	**14.33**	0.23	37.8%	8.8%
6	83.00	**−6827.53**	14230.53	13966.92	13987.99	14029.49	0.0200	0.5273	0.67	--	0.02	36.4%	5.2%

K = number of classes; d = number of parameters; LL = log-likelihood; BIC = Bayesian Information Criterion; SABIC = Sample-size adjusted BIC; CAIC = Consistent Akaike Information Criterion; AWE = Approximate Weight of Evidence Criterion; BLRT = bootstrapped likelihood ratio test; VLMR-LRT = Vuong-Lo–Mendell–Rubin adjusted likelihood ratio test; p = p-value; BF = Bayes Factor; cmP = correct model probability. Bolded values indicate “best” fit for each respective statistic.

##### 2.5.1.2 Descriptive and comparative analyses

Descriptive statistics for each informal caregiver class were calculated using Stata 18.0 (2023). Chi-squares and one-way Analyses of Variance (ANOVAs) were performed to examine differences in all study variables across LCA-identified classes. Table 1 presents descriptive characteristics for the sample and associated bivariate comparisons across informal caregiving motivation classes. These data are provided for context and transparency; however, they are not interpreted as part of the primary analytic results.

#### 2.5.2 Purpose 2: identify demographic and psychological predictors of motivational class membership

A multinomial logistic regression model was estimated in Stata (2023) to examine how informal caregiver and care recipient characteristics, care recipient health challenges, and psychological resources (five subscales and one individual familism item) predicted membership in each motivational class. Supplementary Tables S5 presents standardized coefficients for each psychological resource across motivation class. Full model results with relative risk ratios are provided in Supplementary Tables S6, S7. Results are reported in the text as average marginal effects (AMEs). AMEs express the percentage-point change in the probability of class membership associated with a one-unit change in a predictor (or relative to the reference category for categorical predictors). To highlight findings of practical as well as statistical significance, AMEs with an absolute magnitude of ≥ ± 5 percentage points. This approach provides a clearer interpretation of class-specific demographic and psychological correlates while avoiding the interpretive challenges of comparing odds ratios across separate binary logistic models.

#### 2.5.3 Purpose 3: test informal caregiving motivation classes as moderators of responsibilities and resources

Path models were estimated using Stata’s structural equation modeling (SEM) framework. SEM was chosen for its flexibility in modeling complex relationships among observed variables, its ability to simultaneously test multiple interaction effects, and its capacity to incorporate covariates and multiple outcomes in a single analytic framework. Although no latent constructs were modeled, this approach allowed for the specification of a multivariate system linking informal caregiving stressors, resources, motivation-by-predictor interactions, and wellbeing outcomes.

All continuous variables were standardized prior to model estimation. Interaction terms were constructed by multiplying each standardized informal caregiving stressor or resource variable by binary dummy-coded variables representing each motivation class (reference: situationally motivated). Two path models were estimated for each dependent variable (i.e., standardized acute emotional distress and chronic burnout). Model 1 included informal caregiving motivation classes, informal caregiving stressors, available resources, and interaction terms. Model 2 added informal caregiver and care recipient demographics. This sequential modeling strategy enabled assessment of whether demographic covariates substantively improved model performance and increased variance explained in wellbeing outcomes.

The model was estimated using robust standard errors clustered by motivation class to account for intra-class correlation which can bias standard errors if unaddressed. Because clustering and the inclusion of complex interaction terms preclude the calculation of traditional SEM fit indices (e.g., RMSEA, CFI), model fit was evaluated using the Standardized Root Mean Square Residual (SRMR), with values <0.08 considered acceptable (Hu and Bentler, 1999), and the Coefficient of Determination (CD) to assess total variance explained (StataCorp, 2023). Missing data were handled via listwise deletion, as missingness was minimal (<5% on all variables) and Little’s MCAR test indicated no systematic patterns of missingness.

## 3 Results

### 3.1 Purpose 1: identify distinct informal caregiving motivation classes

**Figure 1:**
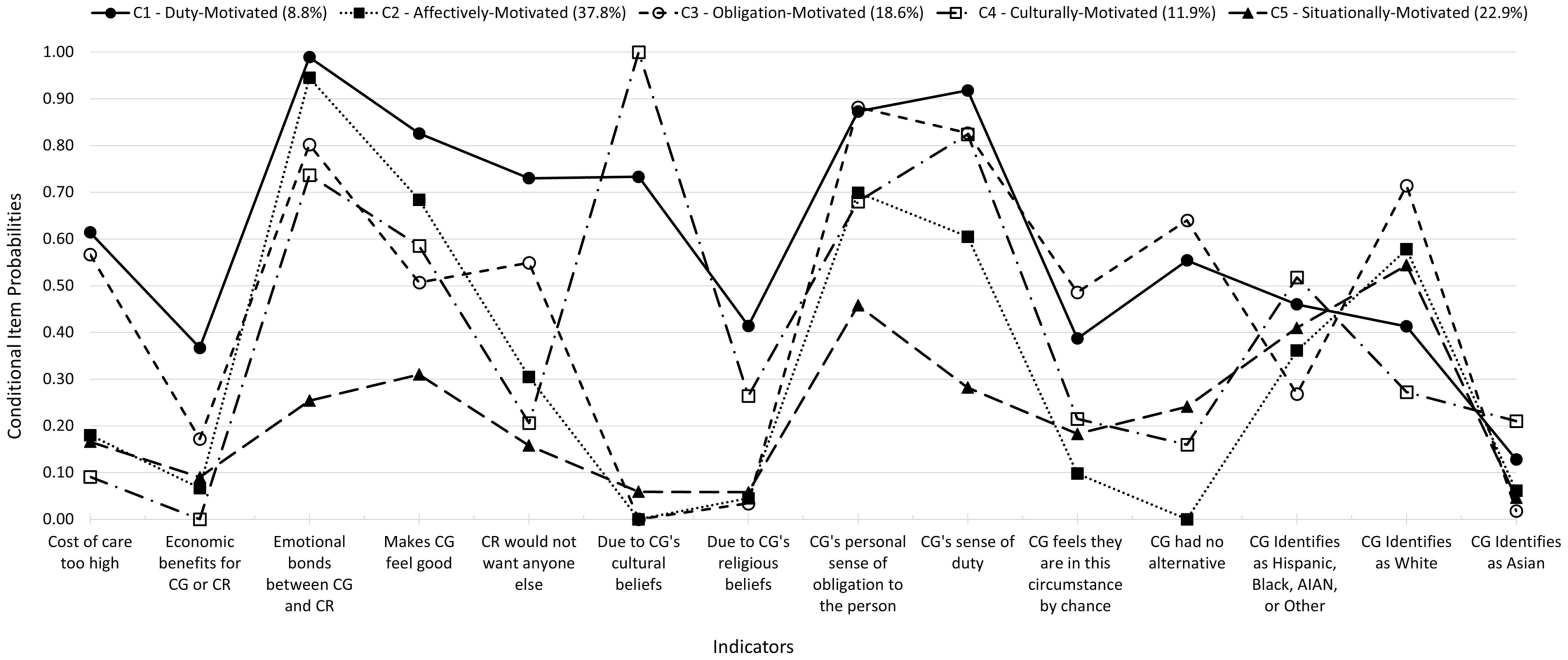
Conditional item probability plot: caregiving motivation classes, caregiver race, and caregiver ethnicity. Class prevalence is in parantheses.

The five-class LCA solution demonstrated the best fit, classification precision, and statistical significance, effectively capturing heterogeneity in informal caregiving motivations. Figure 1 depicts the proportion of class members endorsing each motivation. Classes were labeled based on their unique motivation profiles.

#### 3.1.1 Duty-motivated

Duty-Motivated caregivers (*N* = 90) were defined by high endorsements of duty (91.8%), obligation (87.3%), cultural beliefs (73.3%), and emotional bonds (98.9%). A notable proportion also endorsed religious motivations (41.4%). This class reflects informal caregiving as a moral or cultural responsibility.

#### 3.1.2 Affectively-motivated

Affectively-Motivated caregivers (*N* = 388) were primarily driven by emotional bonds (94.5%) and intrinsic satisfaction (68.4%), with minimal or no endorsement of economic (6.7%), religious (4.5%), and cultural (0%) motivations. These informal caregivers prioritize relational connections over external pressures.

#### 3.1.3 Obligation-motivated

Obligation-Motivated caregivers (*N* = 191) were defined by informal caregiving due to obligation (88.2%), chance circumstances (48.6%), lack of alternatives (64.0%), and financial constraints (56.7%), with moderate emotional investment (80.2%). This class represents informal caregiving driven by necessity rather than choice.

#### 3.1.4 Culturally-motivated

Culturally-Motivated caregivers (*N* = 122) unanimously endorsed cultural motivations (100%), with duty (82.4%) and emotional bonds (73.7%) reinforcing these values.

#### 3.1.5 Situationally-motivated

Situationally-Motivated caregivers (*N* = 235) endorsed fewer motivations overall, with obligation (45.8%) and emotional bonds (25.4%) most frequently cited. This group reflects transient informal caregiving roles influenced by situational factors.

### 3.2 Purpose 2: predict demographic and psychological factors associated with class membership

Analyses of demographic and psychological correlates of informal caregiving class membership were completed using multinomial logistic regression, followed by estimation of average marginal effects (AMEs). AMEs represent the percentage-point change in the predicted probability of membership in a given class for a one-unit change in a continuous predictor or relative to a reference category for categorical predictors, holding other variables constant. Full model results with relative risk ratios are provided in the Supplementary Table S6. Consistent with our focus on practical significance, we highlight predictors with AMEs ≥ ± 5 percentage points, noting that many 95% confidence intervals include zero Supplementary Table S7.

#### 3.2.1 Duty-motivated

Higher probabilities of Duty-Motivated membership were observed for informal caregivers identifying as AIAN (+5.1 percentage points [pp]), Asian (+12.4 pp), another race (+8.5 pp), or Hispanic or Latino (+6.2 pp). Other positive predictors included having the care recipient live with someone else (+6.6 pp), being a spouse/partner (+11.6 pp) or sibling/cousin (+7.4 pp) of the informal caregiver, and caring for someone with a long-term physical condition (+7.3 pp). Lower probabilities were associated with the informal caregiver being female compared to male (−6.6 pp), partnered (−5.0 pp), or caring for someone with an intellectual/developmental disability (−5.2 pp).

#### 3.2.2 Affectively-motivated

Informal caregivers identifying as Black (+5.3 pp), widowed (+7.9 pp), or reporting higher social-support seeking (+6.9 pp) were more likely to be Affectively-Motivated, as were those caring for recipients who lived in a facility (+13.8 pp), had a spouse (+7.5 pp), or were a grandparent (+14.5 pp) or older relative (+13.1 pp). Lower probabilities were linked to identifying as AIAN (−14.0 pp), another race (−16.3 pp), or Hispanic or Latino (−14.1 pp), having stopped working to care for the recipient or currently employed (each −8.4 pp), having the care recipient live with someone (−6.7 pp) or be a child (−13.6 pp), caring for someone with behavioral challenges (−9.5 pp), and reporting higher familism (−5.4 pp).

#### 3.2.3 Obligation-motivated

Female informal caregivers (vs. male) had a higher probability of being Obligation-Motivated (+5.2 pp). Other positive predictors included having stopped working to care for the recipient (+7.7 pp) or being employed (+7.5 pp). Relationship patterns were particularly strong: care recipients who were the informal caregiver’s child (+30.5 pp), sibling/cousin (+19.5 pp), or spouse (+17.2 pp) were much more likely to have Obligation-Motivated informal caregivers, with a smaller increase for parent care (+7.8 pp). Additional positive predictors included having the care recipient live with the informal caregiver (+8.2 pp), and caring for someone with mental health challenges (+7.8 pp), long-term physical conditions (+7.8 pp), or general aging concerns (+5.8 pp). Lower probabilities were associated with identifying as Asian (−17.7 pp), Black (−5.5 pp), Hispanic or Latino (−9.0 pp), or another race (−8.2 pp), being divorced (−5.3 pp) or widowed (−6.3 pp), and having the care recipient live in a facility (−5.0 pp).

#### 3.2.4 Culturally-motivated

Informal caregivers identifying as AIAN (+15.6 pp), Asian (+25.1 pp), Hispanic or Latino (+15.6 pp), Black (+5.7 pp), or another race (+5.8 pp) were more likely to be Culturally-Motivated. Lower probabilities were observed when the care recipient lived with the informal caregiver (−6.1 pp) or was a spouse (−9.9 pp). Caring for someone with behavioral challenges (+7.8 pp) or intellectual/developmental disabilities (+6.8 pp) increased the likelihood of being in this class.

#### 3.2.5 Situationally-motivated

Most associations indicated reduced probabilities of Situationally-Motivated membership. Lower probabilities were linked to identifying as AIAN (−7.7 pp), Asian (−13.4 pp), or Black (−5.2 pp), being widowed (−8.5 pp), or having a female care recipient compared to male (−5.7 pp). Care recipient living situations and relationships were also relevant: living with someone else (−5.5 pp), living in a facility (−5.7 pp), or having a spouse (−5.6 pp), as well as being a spouse (−15.3 pp), parent (−13.3 pp), grandparent (−13.8 pp), older relative (−11.1 pp), child (−13.2 pp), or sibling/cousin (−15.0 pp) of the informal caregiver were linked to lower probabilities. Health conditions associated with reduced probabilities included long-term physical (−11.8 pp), chronic (−6.3 pp), mental health (−7.3 pp), and general aging concerns (−7.5 pp).

### 3.3 Purpose 3: test informal caregiving motivation classes as moderators of responsibilities and resources

Path models were estimated using Stata’s SEM framework to test whether informal caregiving motivation classes moderated the associations among informal caregiving stressors, available resources, and informal caregiver wellbeing. Two versions of each model were computed; one excluding and one including informal caregiver and care recipient demographic covariates. For acute emotional distress, the inclusion of demographic covariates increased variance explained from 17.5 to 35.6%; for chronic burnout, variance explained increased from 25.7 to 29.2%, supporting the value of demographic adjustment in capturing additional explanatory power. Model fit was acceptable for the fully adjusted model (SRMR = 0.012; CD = 0.520). The complete results for both the unadjusted and adjusted models including all main effects and interaction terms are reported in the Supplementary Tables S8, S9. The fully adjusted moderation results are presented in Table 4, which details class-based interactions for informal caregiving stressors and resources across both outcome variables.

**Table 4:** Interaction effects between informal caregiving motivation types and caregiving stressors or resources on adjusted levels of acute emotional distress and chronic burnout.

Variables	Adjusted acute emotional distress				Adjusted chronic burnout			
	β	SE	95th % CI		β	SE	95th % CI	
			Low	High			Low	High
Duty motives by								
Intensive tasks	0.184***	0.003	0.177	0.191	0.225***	0.007	0.212	0.238
Coordination and management tasks	−0.291***	0.021	−0.333	−0.249	−0.344***	0.023	−0.390	−0.298
Caregiving hours/Week	0.035	0.025	−0.013	0.083	−0.053**	0.021	−0.093	−0.013
Caregiving years	−0.121***	0.026	−0.172	−0.070	−0.093***	0.021	−0.134	−0.051
Affective motives by								
Intensive tasks	0.106***	0.008	0.091	0.122	0.146***	0.007	0.133	0.158
Coordination and management tasks	−0.082***	0.014	−0.109	−0.055	−0.227***	0.010	−0.248	−0.207
Caregiving hours/Week	0.101***	0.003	0.095	0.107	0.136***	0.003	0.131	0.142
Caregiving years	0.030	0.019	−0.007	0.067	−0.048***	0.014	−0.075	−0.022
Obligation motives by								
Intensive tasks	0.140***	0.010	0.119	0.160	0.099***	0.010	0.079	0.120
Coordination and management tasks	−0.169***	0.013	−0.194	−0.144	−0.232***	0.007	−0.245	−0.219
Caregiving hours/Week	0.159***	0.007	0.145	0.174	0.041***	0.006	0.029	0.053
Caregiving years	0.073*	0.029	0.016	0.129	−0.007	0.022	−0.050	0.035
Culture motives by								
Intensive tasks	0.026***	0.006	0.014	0.038	0.164***	0.014	0.136	0.191
Coordination and management tasks	0.055**	0.017	0.021	0.089	−0.129***	0.014	−0.156	−0.101
Caregiving hours/Week	0.043***	0.007	0.028	0.058	0.048***	0.007	0.034	0.062
Caregiving years	−0.085***	0.013	−0.112	−0.059	−0.059***	0.008	−0.074	−0.043
Social support by								
Duty motives	0.057	0.042	−0.025	0.139	0.143*	0.063	0.019	0.267
Affective motives	0.025	0.039	−0.050	0.101	0.055	0.069	−0.079	0.189
Obligation motives	−0.004	0.043	−0.088	0.080	0.025	0.069	−0.110	0.160
Culture motives	0.114*	0.045	0.025	0.202	0.065	0.064	−0.061	0.191
Resilience x								
Duty motives	−0.139***	0.026	−0.191	−0.088	−0.108**	0.038	−0.182	−0.035
Affective motives	−0.178***	0.028	−0.233	−0.123	−0.109*	0.044	−0.194	−0.023
Obligation motives	−0.077***	0.020	−0.116	−0.038	−0.107**	0.035	−0.176	−0.038
Culture motives	−0.216***	0.032	−0.279	−0.153	−0.122***	0.036	−0.192	−0.052

*p < 0.05; **p < 0.01; **p < 0.001; Standardized regression coefficients (β), standard errors (SE), and 95% confidence intervals (CI) are presented for each interaction term. Models include demographic covariates and main effects for all predictors (see Supplementary Tables S7, S8 for all variables). Bolded coefficients denote statistically significant interactions.

#### 3.3.1 Overall effects

Affectively-Motivated informal caregivers reported higher acute emotional distress (*β* = 0.051) but significantly lower chronic burnout (*β* = −0.063). Obligation-Motivated and Culturally-Motivated informal caregivers experienced elevated levels of both distress (Obligation: *β* = 0.168; Culture: *β* = 0.155) and burnout (Obligation: *β* = 0.101; Culture: *β* = 0.066). Duty-Motivated informal caregivers did not differ significantly from the reference group in either outcome.

Resilience and social support consistently predicted better outcomes. Greater resilience was associated with lower distress (*β* = −0.137) and burnout (*β* = −0.204). Social support similarly predicted reduced distress (*β* = −0.169) and burnout (*β* = −0.242). These effects remained robust across both models.

Regarding objective informal caregiving stressors, more hours per week providing informal care predicted increased burnout (*β* = −0.042) but not distress. Years of informal caregiving was positively associated with burnout (*β* = 0.032) but showed no significant relationship with acute distress. Task complexity also contributed meaningfully: greater involvement in direct care and coordination/management tasks was associated with higher levels of both outcomes. Coordination and management responsibilities in particular predicted elevated distress (*β* = 0.123) and burnout (*β* = 0.247).

#### 3.3.2 Moderation of stressors

##### 3.3.2.1 Intensive personal care tasks

Increased involvement in direct care tasks predicted higher distress and burnout across all classes. These effects were especially pronounced for Duty-Motivated informal caregivers (distress: *β* = 0.184; burnout: *β* = 0.225). Affectively-, Obligation-, and Culturally-Motivated informal caregivers also showed significant increases in both outcomes as direct task involvement increased.

##### 3.3.2.2 Coordination and management tasks

For most groups, coordination tasks were positively associated with both outcomes. However, the strength of the association varied. Obligation-Motivated (distress: *β* = −0.169; burnout: *β* = −0.232) and Affectively-Motivated informal caregivers (distress: β = −0.082; burnout: β = −0.227) showed attenuated burnout, suggesting that for some, coordination work may foster control or structure that buffers emotional exhaustion. Culturally-Motivated informal caregivers, by contrast, reported increased distress (*β* = 0.055) in response to coordination demands, while still benefiting from lower burnout (*β* = −0.129).

##### 3.3.2.3 Informal caregiving hours

The number of hours spent with informal caregiving was significantly associated with greater distress and burnout among Affectively-, Obligation-, and Culturally-Motivated informal caregivers. Among Duty-Motivated informal caregivers, hours predicted lower burnout (*β* = −0.053) but were unrelated to distress.

##### 3.3.2.4 Years of care

Duration of informal caregiving exhibited differential effects across groups. Longer care durations predicted lower distress and burnout among Duty-Motivated (distress: *β* = −0.121; burnout: *β* = −0.093) and Culturally-Motivated informal caregivers (distress: *β* = −0.085; burnout: *β* = −0.059). In contrast, Obligation-Motivated informal caregivers reported increased distress (*β* = 0.073), with no significant effect on burnout.

#### 3.3.3 Moderation of resources

##### 3.3.3.1 Resilience

Consistent protective effects were observed for resilience across all classes. The strongest effect on distress occurred among Culturally-Motivated informal caregivers (*β* = −0.216), followed by Affectively-Motivated (*β* = −0.178), Duty-Motivated (*β* = −0.139), and Obligation-Motivated informal caregivers (*β* = −0.077). Similar patterns emerged for burnout, with resilience buffering exhaustion most strongly among Culturally-Motivated (*β* = −0.122) and Affectively-Motivated (*β* = −0.109) groups.

##### 3.3.3.2 Social support

Social support was less consistently protective across classes. For most groups, support did not significantly moderate distress or burnout. However, among Culturally-Motivated informal caregivers, higher social support was paradoxically associated with increased acute distress (*β* = 0.114, *p* = 0.012).

## 4 Discussion

This study applied the ICIM (Gérain and Zech, 2019) to examine how informal caregiving motivations moderate associations among informal caregiving stressors, resources, and informal caregiver wellbeing. Using a large, demographically diverse sample and a multidimensional set of predictors, the study addressed three interconnected research purposes: identifying informal caregiver motivation profiles, evaluating their demographic and psychological correlates, and testing whether these profiles moderated the effects of objective informal caregiving burdens and resource availability on acute psychological distress and burnout. Results affirmed that motivations are both structurally embedded and psychologically consequential, influencing how informal caregivers assume their roles as well as how they experience and adapt to informal caregiving stress. Further, across study purposes, findings support a core ICIM proposition that outcomes emerge from the fit between stressor exposure and the informal caregiver’s appraisal context, further shaped by resources and sociocultural structures.

### 4.1 Purposes 1 and 2: profiles and predictors of informal caregiving motivation classes

LCA identified five distinct motivation profiles: Duty-, Affectively-, Obligation-, Culturally-, and Situationally-Motivated. These profiles move beyond the traditional intrinsic-extrinsic dichotomy (Deci and Ryan, 2008) to capture the complex interplay of personal values, relational obligations, cultural scripts, and situational constraints that give informal caregiving its meaning. Consistent with the ICIM (Gérain and Zech, 2019), the classes reflect varied internalization pathways, from highly autonomous to highly controlled, and from strongly identity-integrated to weakly internalized.

Subsequent analyses examined how demographic, relational, and health-related characteristics differentiated these classes, exposing how informal caregiver orientations are embedded in sociocultural contexts and shaped by intersecting personal and structural factors. For each profile, the motivational content, key predictors, and potential psychological mechanisms linking motivation to stressor–resource dynamics are considered together. To further differentiate profiles beyond the composite resilience and social support indicators used in later analyses, resource patterns are also described at the subscale level (i.e., self-esteem, mastery, optimism, spirituality/religiosity, familism, social-support seeking). Taken as a whole, these results support ICIM’s premise that the meaning informal caregivers attach to their role is not static; rather, it is maintained or eroded through appraisal processes, resource alignment, and the broader sociocultural environment (Gérain and Zech, 2019).

#### 4.1.1 Duty-motivated

Duty-Motivated informal caregivers reported high endorsement of duty/obligation motives (e.g., personal sense of responsibility, moral duty) and cultural/religious beliefs (e.g., acting in accordance with cultural norms, fulfilling religious obligations) alongside moderate endorsement of emotional bonds. These patterns are consistent with collectivist ideologies like filial piety (Pan et al., 2022) and moral obligation (Knight and Sayegh, 2010). This constellation of motives frames informal caregiving as a moral imperative and a central element of personal and family identity. In ICIM terms, this represents a highly integrated internalization pathway, where informal caregiving is appraised as both identity-consistent and value-congruent (Gérain and Zech, 2019, 2022). Such appraisals can sustain long-term commitment under demanding conditions and orient stress appraisals toward challenge rather than threat, supporting persistent engagement and lowering the risk of disengagement (Wang et al., 2020).

Consistent with this interpretation, Duty-Motivated informal caregivers were disproportionately male, more often identified as AIAN, Asian, Hispanic or Latino, or another race, and frequently cared for close kin, especially spouses or siblings/cousins, who often lived separately from them. They were also more likely to care for someone with a long-term physical condition. In contrast, partnered informal caregivers and those caring for individuals with intellectual/developmental disabilities were less represented, possibly reflecting competing household role demands or different task-allocation patterns.

Psychological and social resource patterns for this class showed high self-esteem, mastery, optimism, and spirituality/religiosity, indicating a robust internal resource base. These personal resources are consistent with ICIM’s proposition that identity-congruent appraisals facilitate mobilization of coping strengths. However, this group reported low social-support seeking, which may indicate reluctance to rely on help outside the family, a pattern also observed in other duty-driven contexts. Such reluctance can limit the benefits of available supports, particularly when external assistance is perceived as challenging cultural norms or personal responsibility (Kong et al., 2021; Wittenberg-Lyles et al., 2013).

The costs of caring in duty-bound contexts are, therefore, two-fold. While internalized duty and moral responsibility can promote persistence, meaning-focused coping, and resilience in the face of chronic demands (Luthans et al., 2007; Palacio et al., 2020), they may also heighten susceptibility to guilt, self-criticism, and perceived role failure if external resources are scarce or misaligned with values (Bevans and Sternberg, 2012). In ICIM terms, the fit between appraisal context and resource environment becomes critical: congruence can enhance adaptation while misalignment can transform potentially helpful supports into sources of tension and strain (Gérain and Zech, 2019, 2022).

#### 4.1.2 Affectively-motivated

Affectively-Motivated informal caregivers endorsed high levels of emotional bond motives (e.g., love, affection, mutual preference for the informal caregiving relationship) and low endorsement of duty/obligation, cultural, or constraint-related motives. This profile reflects voluntary, identity-consistent informal caregiving in which the role aligns closely with personal values and relational commitments (Greenwood and Smith, 2019; Quinn et al., 2010). Within ICIM, these represent an autonomous internalization pathway, where informal caregiving is appraised as an expression of self-chosen goals and meaningful interpersonal connections (Gérain and Zech, 2019, 2022). Such appraisals often promote approach-oriented coping, cultivate sustained engagement, and may protect against disengagement, particularly when the role is a source of mutual satisfaction (Hoyt et al., 2024).

Affectively-Motivated informal caregivers were more likely to be Black or widowed and to care for recipients living in facilities, with spouses, or who were grandparents or older relatives. They were less likely to identify as AIAN, Hispanic or Latino, or another race, and less likely to care for children or individuals with behavioral challenges. These patterns suggest an informal caregiving context shaped by enduring, emotionally close relationships, often outside the immediate household, and by later-life transitions such as widowhood, where informal caregiving may help maintain social connection and purpose.

Psychological and social resource patterns indicate moderate optimism, average self-esteem and mastery, and relatively high social-support seeking compared with other classes. Notably, familism scores were lower than in other intrinsically oriented classes, suggesting engagement is driven more by emotional closeness than by cultural obligation. In ICIM terms, reliance on relational coping can enhance informal caregiving’s positive meaning, yet these same ties may intensify distress if relationships are threatened by decline, conflict, or loss (Gérain and Zech, 2019; Roth et al., 2018).

Psychologically, the same emotional investment that sustains informal caregiving may heighten role engulfment and empathic strain when care demands escalate (Skaff and Pearlin, 1992). Mechanisms such as blurred boundaries, hyper-responsiveness to recipient distress, and over-identification with the informal caregiving may help explain why these informal caregivers can be resilient under moderate demands but vulnerable when stressors are high (Schulz and Eden, 2016; Beach et al., 2022). ICIM’s dual-process proposition that appraisal quality determines whether stressors are experienced as fulfilling challenges or depleting threats is especially relevant here (Gérain and Zech, 2019). The prominence of widowhood in this class may also reflect post-loss reorientation toward informal caregiving as a meaningful role, coupled with increased risk of grief-related stress and anticipatory loss (Aneshensel and Mitchell, 2014).

#### 4.1.3 Obligation-motivated

Obligation-Motivated informal caregivers endorsed high levels of constraint and external determination motives including having no alternative, caring by chance, or avoiding the cost of professional care, paired with moderate duty but lower emotional bond or cultural/religious endorsement. This constellation suggests necessity-driven informal care, often undertaken under conditions of financial strain, limited alternatives, or situational coercion. Within the ICIM framework, these informal caregivers exemplify a controlled internalization pathway, where informal caregiving is appraised as externally imposed rather than self-endorsed (Gérain and Zech, 2019, 2022). Such low-choice appraisals are consistently linked to higher perceived burden, reduced coping flexibility, and diminished capacity to mobilize internal resources (Chappell and Funk, 2011; Losada et al., 2010).

Obligation-Motivated informal caregivers were more likely to be female, employed or to have stopped working to provide care, and to care for close kin, especially children, siblings/cousins, or spouses, who often live with them. They were also more likely to care for recipients with mental health challenges, long-term physical conditions, or aging-related concerns. In contrast, membership likelihood was lower among informal caregivers identifying as Asian, Black, Hispanic or Latino, or another race, and among those who were divorced, widowed, or caring for recipients in facilities.

Psychological and social resource patterns for this class showed low self-esteem, low optimism, and low spirituality/religiosity compared with more intrinsically motivated classes, with slightly above-average mastery but notably low social-support seeking. This pattern suggests that although some task management competence may be present, informal caregivers in this class may lack the relational coping orientation and personal efficacy beliefs that buffer stress in other groups. In ICIM terms, the mismatch between constrained motivation and limited internal resources likely encourages appraisals of informal caregiving demands as threats rather than challenges (Gérain and Zech, 2019, 2022).

Psychological mechanisms in this group may include resentment toward the informal caregiving role, perceived entrapment, and helplessness, all of which can erode motivation over time and contribute to emotional exhaustion (Zarit and Edwards, 2008; Quinn et al., 2010). The co-occurrence of employment and informal caregiving may further amplify time strain, reinforcing the external-control dimension of this class. ICIM predicts that when motivation is driven by external pressure rather than personal endorsement, even potentially protective resources such as social support or resilience may have attenuated or null effects, and, in some cases, may exacerbate stress if perceived as intrusive or judgmental (Bevans and Sternberg, 2012; Gérain and Zech, 2019, 2022).

#### 4.1.4 Culturally-motivated

Culturally-Motivated informal caregivers grounded their informal caregiving meaning in cultural identity and spiritual belief, with high endorsement of motives related to both. In the ICIM framework, this profile embodies a highly integrated internalization pathway in which appraisals are anchored in culturally endorsed values (Gérain and Zech, 2019, 2022). Such motives can be protective when the informal caregiving context is culturally congruent, reinforcing role legitimacy, affirming identity, and sustaining engagement through meaning-focused coping (Mehdipanah et al., 2024).

Culturally-Motivated informal caregivers were more likely to be AIAN, Asian, Hispanic or Latino, Black, or another race, and to care for recipients with behavioral or intellectual/developmental disabilities. They were less likely to live with the care recipient or to care for a spouse. These patterns are consistent with traditional role distributions in multigenerational households, where informal caregiving responsibilities are shared, segmented, or assigned based on kinship hierarchy and gender norms (Dilworth-Anderson et al., 2002; Miyawaki, 2016).

Psychological and social resource patterns indicated high spirituality/religiosity, high familism, and moderately high social-support seeking, alongside modest self-esteem and optimism. This pattern suggests that resilience in this group is rooted more in cultural and relational values rather than in personal efficacy beliefs. From an ICIM perspective, the fit between appraisal context and environmental inputs is especially critical: consistency between services and cultural expectations can strengthen adaptation while misalignment can function as a secondary stressor, reframing otherwise supportive inputs as intrusive, disrespectful, or even harmful (Gérain and Zech, 2019, 2022; Zarzycki et al., 2022, 2023).

Psychological mechanisms likely operating here are three-fold. First, identity affirmation occurs when informal caregiving aligns with cultural and spiritual values, promoting persistence even under high demands (Luthans et al., 2007; Palacio et al., 2020). Second, cultural dissonance arises when formal care systems fail to respect or accommodate cultural scripts, eroding trust and perceived role legitimacy (Hickson, 2022). Third, reciprocity norms, where informal caregiving is embedded in a moral economy of mutual support, can make withdrawal less likely but also more vulnerable to guilt when obligations cannot be met (Prunty and Foli, 2019). In ICIM terms, meaning-making through cultural scripts can serve as a powerful resilience resource, but only when environmental inputs reinforce rather than undermine that meaning (Gérain and Zech, 2019, 2022). Misaligned systems may not only fail to assist, they may also actively produce strain by threatening a informal caregiver’s role identity (Gérain and Zech, 2022; Prunty and Foli, 2019).

#### 4.1.5 Situationally-motivated

Situationally-Motivated informal caregivers showed low endorsement across all motivation dimensions, indicating episodic, circumstantial, or transitional informal caregiving roles with minimal identity salience. This is consistent with Zarzycki et al.’s (2023) pragmatic informal caregivers, who assume the role due to immediate need, crisis, or diffuse family expectation but do not integrate informal caregiving into their self-concept. In ICIM terms, this profile represents a weak or absent internalization pathway (Gérain and Zech, 2019, 2022). Informal caregiving is appraised as a temporary or externally imposed task rather than as a meaningful role, leaving few stable motivational anchors to guide coping. Such low-salience appraisals can produce fragile meaning structures, in which stressor–resource dynamics are highly context-dependent (Gérain and Zech, 2022; Zarzycki and Morrison, 2021).

Demographically, these informal caregivers were less likely to be AIAN, Asian, or Black, less likely to be widowed, and less likely to care for female recipients, co-resident recipients, or those in facilities. They were also less likely to occupy close familial roles like spouse, parent, grandparent, or sibling/cousin. Health-related predictors including long-term physical, chronic, mental health, and aging-related conditions were uniformly associated with lower probability of membership, suggesting that situational informal caregiving often involves shorter-term, lower-complexity cases.

Psychological and social resource patterns for this class reinforce this representation. Situationally-Motivated informal caregivers scored lowest on self-esteem and mastery, with no compensating strengths in optimism, familism, or spirituality/religiosity. While they demonstrated slightly higher social-support seeking than some other low-resource groups, the absence of a coherent value or identity framework may limit their ability to mobilize and sustain those supports effectively.

Psychological mechanisms likely operating in this class include low role preparedness and minimal role commitment, both of which can undermine sustained engagement in informal caregiving responsibilities (Dal Pizzol et al., 2024; Hancock et al., 2022). These informal caregivers may also exhibit reduced anticipatory coping capacity, making their responses to stress more reactive than proactive (Sołtys and Wnuk, 2025). In addition, the weak integration of informal caregiving into their self-concept may leave them more prone to disengagement when demands escalate or when available supports are insufficient (Beach et al., 2022; Sołtys and Wnuk, 2025). In the ICIM framework, the absence of a strong appraisal orientation is itself a risk factor; that is, without an internalized meaning system to buffer challenges, informal caregiving outcomes are more vulnerable to contextual instability (Gérain and Zech, 2019, 2022). Even potentially protective resources (e.g., social support) may be underutilized or inconsistently applied, resulting in greater variability in wellbeing (Beach et al., 2022).

#### 4.1.6 Purposes 1 and 2 conclusion

Collectively, these five informal caregiver motivation classes map onto distinct internalization pathways in the ICIM framework (Gérain and Zech, 2019, 2022), ranging from highly autonomous (Affectively-Motivated) to highly controlled (Obligation-Motivated) or weakly internalized (Situationally-Motivated). Each pathway carries its own constellation of psychological tensions, like guilt, role engulfment, perceived coercion, cultural dissonance, and low identity salience, that function as mechanisms linking stressor exposure to wellbeing outcomes. These mechanisms explain why informal caregivers facing similar objective demands can diverge sharply in coping strategies, resource use, and long-term adjustment.

The profiles also demonstrate that motivation is not solely an individual disposition but emerges at the intersection of personal identity, relationship context, and cultural norms. Demographic and relational configurations including kinship role, co-residence, racial/ethnic identity, and recipient health status affect how informal caregiving is appraised which, in turn, influences whether stressors are viewed as challenges to be met or as threats to be endured. Psychological and social resource patterns, particularly the composition of resilience (e.g., self-esteem, mastery, optimism) and social support (e.g., familism, spirituality/religiosity, support-seeking), further differentiate classes, demonstrating that resilience is not a monolith; instead, it embodies a set of components that may align or misalign with specific motivational orientations.

### 4.2 Purpose 3: test informal caregiving motivation classes as moderators of responsibilities and resources

Consistent with the ICIM, informal caregiving motivations moderated the effects of objective informal caregiving burdens, resource availability, and both acute emotional distress and chronic burnout. Importantly, these outcomes were related but not redundant; that is, several task-related predictors and class interactions diverged across the two outcomes, indicating ICIM’s distinction between immediate affective strain and longer-term depletion, and emphasizing the need for differentiated intervention strategies (Gérain and Zech, 2021). Situationally-Motivated informal caregivers served as the reference group as their low motivational salience provides a useful baseline for interpreting how informal caregiving meaning shapes stressor–resource responses.

#### 4.2.1 Task-related stressors

Duty-Motivated informal caregivers experienced heightened distress and burnout with greater involvement in personal care tasks but reduced strain when engaged in coordination and management, aligning with their strong preference for structured, identity-congruent roles described in Purpose 2 (Bevans and Sternberg, 2012). Affectively-Motivated informal caregivers showed a similar split. Specifically, emotional investment buffered the impact of coordination tasks but amplified reactivity to personal care demands (Pinquart and Sörensen, 2003). For Obligation-Motivated informal caregivers, high distress and burnout persisted across all task types, with only modest relief in burnout for coordination work, consistent with their lower internal resources and externally controlled appraisals (Chappell and Funk, 2011). Culturally-Motivated informal caregivers displayed a distinctive pattern; coordination reduced burnout but increased distress, likely reflecting dissonance between cultural values and bureaucratic care processes (Mehdipanah et al., 2024).

#### 4.2.2 Duration and intensity

Weekly informal caregiving hours predicted greater distress and burnout among Affectively-, Obligation-, and Culturally-Motivated informal caregivers, but not among Duty-Motivated informal caregivers. For the latter group, more hours reduced burnout, consistent with role alignment reinforcing purpose (Gérain and Zech, 2019). Years of informal caregiving differentiated outcomes. Longer informal caregiving predicted reduced distress and burnout among Duty- and Culturally-Motivated informal caregivers, but increased distress for Obligation-Motivated informal caregivers. Affectively-Motivated informal caregivers experienced declining burnout over time, suggesting emotional commitment supports sustained engagement without necessarily protecting against acute reactivity.

#### 4.2.3 Resources as moderators

Resilience was protective across all classes, but both its magnitude and relative ranking differed for acute distress versus burnout. For acute distress, the strongest buffering occurred among Culturally-Motivated informal caregivers, followed by Affectively-Motivated, Duty-Motivated, and Obligation-Motivated informal caregivers. For burnout, resilience again had the largest effect among Culturally-Motivated informal caregivers, but the second strongest buffering shifted to Obligation-Motivated informal caregivers, followed closely by Affectively-Motivated and Duty-Motivated informal caregivers. This shift, particularly the rise of the Obligation-Motivated group for burnout, suggests that resilience may operate through somewhat different mechanisms for immediate emotional strain versus longer-term depletion. Informal caregivers with more internalized or identity-based motivations generally appeared to mobilize internal resources like mastery and self-efficacy more effectively than those driven by other motives (Allen et al., 2021; Barry et al., 2021). In ICIM terms, acute distress may be more sensitive to the alignment between personal meaning systems and short-term coping capacity, whereas burnout may be more affected by sustained role endurance and the capacity to deploy resources over time (Gérain and Zech, 2019, 2022). These acute–chronic divergences emphasize the need to tailor interventions not only to motivational profiles but also to the temporal dynamics of strain. This distinction sets the stage for identifying which strategies may yield immediate relief versus those that preserve engagement and prevent depletion over the long term.

By contrast, social support showed only two significant moderation effects, both of which diverged in direction from the protective pattern seen for resilience. Among Duty-Motivated informal caregivers, higher social support predicted higher burnout, potentially reflecting guilt or perceived role dilution when internalized responsibility norms are challenged by accepting outside help (Knight and Sayegh, 2010). Among Culturally-Motivated informal caregivers, higher social support was associated with greater acute distress, perhaps reflecting dissonance between cultural norms of family care and the use of formal or external supports (Chappell and Funk, 2011; Palacio et al., 2020). No significant moderation effects emerged for Affectively- or Obligation-Motivated informal caregivers. These paradoxical patterns reinforce that resources are not universally beneficial; their effects depend on alignment with motivational orientation and appraisal context, further emphasizing the need for intervention designs that are both context- and meaning-sensitive.

#### 4.2.4 Model comparisons

Adding demographic covariates doubled the variance explained in acute distress and modestly increased variance explained for burnout. The demographic-adjusted models retained the core interaction patterns observed in the simpler specifications, though the magnitude of several effects shifted and a few lost or gained significance. These shifts suggest the importance of accounting for sociodemographic context when modeling stress processes in informal caregiving populations.

#### 4.2.5 Purpose 3 conclusion

Together, these findings demonstrate that the interplay of stressors, resources, and informal caregiving meaning is highly profile-specific and outcome-dependent. While resilience emerged as a broadly protective factor, its effects varied by both motivational class and whether the outcome reflected acute emotional strain or longer-term burnout. Intervention strategies must therefore consider not only the type of stressor or resource but also the informal caregiver’s motivational orientation and the temporal horizon of the intended impact.

### 4.3 Intervention implications: practice- and program-level recommendations

This study highlights the need for motivation-aligned supports that address both what informal caregivers do and why they do it. The impact of stressors and resources depends on the informal caregiving tasks involved and the motivational lens through which those tasks are interpreted. Interventions should, therefore, be differentiated across profiles. Duty-Motivated informal caregivers, whose roles are anchored in moral obligation, spiritual belief, and cultural expectations, may benefit from values-based and mindfulness approaches that sustain meaning while addressing guilt or overextension (Allen et al., 2021; Ozcan et al., 2021). Affectively-Motivated informal caregivers, for whom emotional closeness is both a strength and a source of vulnerability, may respond well to emotion regulation training and peer-based support to help set boundaries and reduce reactivity to high-intensity tasks (Allen et al., 2021; Cooper et al., 2024). Obligation-Motivated informal caregivers, often operating under external pressures, may require interventions that reduce coercion and restore agency, such as workplace flexibility programs, financial counseling, and informal caregiver rights education (Lee et al., 2024; Dombestein et al., 2020). Culturally-Motivated informal caregivers may benefit from services that are explicitly culturally congruent, such as faith-based initiatives, language-accessible resources, or culturally grounded care navigation (Knight and Sayegh, 2010; Zarzycki et al., 2022). Finally, Situationally-Motivated informal caregivers, whose roles lack strong identity anchoring, may need flexible supports including transitional counseling, temporary respite, and early guidance to clarify expectations and promote role preparedness (Barry et al., 2021; Wylie et al., 2021).

### 4.4 Policy implications: macro-level recommendations

At the systems level, integrating brief motivational screening into informal caregiver intake could guide resource allocation and ensure supports are tailored from the outset. Such screening could inform priority-setting in public health agencies, health systems, and community organizations; for example, channeling workplace flexibility and financial assistance toward Obligation-Motivated informal caregivers, culturally tailored navigation services toward Culturally-Motivated informal caregivers, and transitional supports toward Situationally-Motivated informal caregivers. Embedding these differentiated approaches into informal caregiver support legislation, respite funding formulas, and provider training standards would enhance uptake, effectiveness, and equity. More broadly, aligning policy incentives and service delivery models with informal caregivers’ motivational orientations may improve intervention adherence, extend informal caregiver role sustainability, and reduce the societal costs of informal caregiver burnout.

## 5 Conclusion: strengths, limitations, and future directions

This study offers a novel and empirically grounded application of the ICIM by incorporating informal caregiving motivation into multivariate models of objective burden, psychosocial resources, and informal caregiver wellbeing. Central strengths of this work lie in its large and demographically diverse sample and its use of LCA to capture real-world constellations of informal caregiving motives, an advance beyond traditional variable-centered models. The inclusion of culturally relevant motivational items, derived from EUROFAMCARE Consortium (2006), allowed for a differentiated, context-sensitive understanding of informal caregiving roles.

Several limitations should be noted. First, the present study used a person-centered approach (LCA) to identify discrete informal caregiving motivation profiles. While this approach has clear advantages for capturing naturally occurring combinations of motives, it is inherently individual-centered and does not provide direct estimates of variable-level relationships that can be generalized across all informal caregivers. Future research should integrate variable-centered analyses (e.g., regression, structural equation modeling with latent variables, or longitudinal cross-lagged models) with person-centered typologies to examine both between-person heterogeneity and within-variable associations. Combining these approaches could offer a more comprehensive understanding of how specific motivational dimensions operate within and across informal caregiver subgroups. Second, the cross-sectional design precludes causal inference, and motivations may shift over time as stressors, resources, and role identities change. While some core elements of motivation like enduring values or deeply held cultural beliefs may remain relatively stable, other aspects may be more situationally responsive, adapting to changing informal caregiving demands, health trajectories, or the availability of supports. Longitudinal studies that track both stability and change in motivational configurations, alongside wellbeing outcomes, would strengthen inferences about directionality and clarify the conditions under which motivations are more malleable versus enduring. Finally, although the sample included informal caregivers diverse in race and ethnicity, geographic location, and informal caregiving contexts, replication in international or more socioeconomically varied samples is warranted, as cultural norms and policy supports may influence both the composition and the implications of informal caregiving motivation profiles.

In practice, this work substantiates the importance of considering informal caregiving motivations and cultural contexts when investigating the influence of stressors, resources, and wellbeing. Informal caregiving is more than a set of tasks; rather, it is a complex, value-laden experience embedded in multiple individual and intersecting personal, cultural, and structural contexts. Recognizing motivational diversity and tailoring supports to this diversity is likely necessary for reducing burnout, improving quality of informal caregiving, and sustaining informal caregiving as a vital component of aging societies (Gérain and Zech, 2021).

## Data Availability

The data are available upon reasonable request for purposes of verification or meta-analyses. The full dataset is not publicly available due to ongoing analyses for future publications. Please contact Dr. Deborah Nichols for more information regarding data access.
