# Supplementary material for: Motivated to care: latent classes of caregiver motivation as moderators among stress, resources, and well-being
Source: Front Psychol. 2025 Nov 12;16:1608435. doi: 10.3389/fpsyg.2025.1608435 (PMC12646912; doi:10.3389/fpsyg.2025.1608435)
Supplement: Supplementary file 1 [file Data_Sheet_1.pdf]

APPENDIX

- Factor Analysis of Informal Caregiving Activities
- **Table S1.** Tetrachoric Correlation Matrix of All Binary Informal Caregiving Activities
- **Table S2.** Factors and Eigenvalues
- **Table S3.** Factor Loadings, Uniqueness, and Communalities for Informal Caregiving Activities
- **Table S4.** Factor Rotation Matrix
- **Table S5.** Resilience and Social Support Resources Split into Subscales across Informal Caregiver Motivation Class
- **Table S6.** Multinomial Logistic Regression Predicting Class Membership from Demographics, Care Recipient's Health Challenges, and Informal Caregiver Resources
- **Table S7.** Average Marginal Effects Associated with the Multinomial Logistic Regression Predicting Class Membership from Demographics, Health Challenges of the Person Needing Care, and Informal Caregiver Resources
- **Table S8.** Unadjusted and Adjusted Predictors of Acute Emotional Distress Across Informal Caregiving Motivation Profiles
- **Table S9.** Unadjusted and Adjusted Predictors of Chronic Burnout Across Informal Caregiving Motivation Profiles

Factor Analysis of Informal Caregiving Activities

Informal caregivers were asked to select all activities they assisted the person needing care with (i.e., yes/no). These activities included five Activities of Daily Living (ADLs), six Instrumental Activities of Daily Living (IADLs), and eight medical and nursing tasks. The analysis completed using Stata v.18.0 is outlined below.

1. First, a tetrachoric correlation matrix was computed to assess associations among the binary informal caregiving activity variables. Because one negative eigenvalue was detected, the matrix was adjusted to be positive semidefinite. These correlations revealed meaningful patterns of co-occurrence among caregiving tasks and are reported in Table S1.
2. Next, a factor analysis, using the iterated principal factors method, two factors were retained based on eigenvalues greater than 1, interpretability, and inspection of the scree plot. The first factor had a substantial eigenvalue of 6.90, indicating it explained a large proportion of shared variance. Factor loadings were extracted and interpreted after rotation. Loadings greater than |0.30| were considered meaningful indicators of a factor. Results are shown in Table S2.
3. To facilitate interpretation, an oblique ProMax rotation was applied to the factors. This rotation allowed for correlation between factors, reflecting the real, world complexity of informal caregiving activities. The rotation matrix indicated how the original factors are related to the rotated factors. The proportion of variance explained by each factor was substantial, with Factor 1 accounting for the most variance (77.6%) followed by Factor 2 (53.7%). The unique variances for each variable suggested a decent level of specificity, although giving medicines shared common variance as indicated by its loadings on two factors.
4. Uniqueness values (1- communality) represent the proportion of variance in each informal caregiving activity variable that is not explained by the retained factors. While most activities were well represented, variables with higher uniqueness (e.g., service coordination) may reflect either more situationally variable tasks or measurement error.

Composite scores for each factor were calculated by adding the number of activities associated with each factor together. Reliability estimates were computed using Kuder Richardson 20, an estimate appropriate for dichotomous variables. Each factor is described below:

- Factor 1, **Intensive Personal Care**, loads highly on tasks that involve hands-on, physical caregiving and personal assistance. Tasks include toileting, dressing, bathing, bed/chair transfers, feeding, incontinence care, meal prep, wound care, medication assistance, and medical equipment use. These tasks require close personal contact, often involve physical labor or medical knowledge, and are closely associated with Activities of Daily Living (ADLs) and instrumental health support. Kuder- Richardson (KR-20) = 0.79.
- Factor 2, **Coordination and Household Management**, loads on items that reflect logistical, financial, and household responsibilities, often falling under Instrumental Activities of Daily Living (IADLs) and broader care management duties. High-loading items include blood pressure and blood glucose monitoring, transportation, grocery and other shopping, managing finances, service coordination, and preparation of special meals. These tasks reflect indirect care, including managing appointments, food, money, and medical monitoring, work often invisible but essential.. The internal consistency of this factor is indicated by a KR-20 = 0.61.

Table S1.

Tetrachoric Correlation Matrix of All Binary informal Caregiving Activities

	1	2	3	4	5	6	7	8	9	10	11	12	13	14	15	16	17	18
1. Transportation	1.0																	
2. Grocery shopping	**.35**	1.0																
3. Housework	**.12**	**.49**	1.0															
4. Prepare meals	**.24**	**.49**	**.55**	1.0														
5. Manage finances	**.20**	**.18**	-.01	.07	1.0													
6. Arrange services	**.35**	**.20**	.01	**.10**	**.43**	1.0												
7. Get in/out of beds/chairs	**.34**	**.20**	**.32**	**.39**	-.02	**.17**	1.0											
8. Get dressed	**.32**	**.26**	**.23**	**.51**	.10	**.20**	**.69**	1.0										
9. Get to/from toilet	**.29**	**.19**	**.34**	**.48**	.08	**.26**	**.77**	**.70**	1.0									
10. Bathe or shower	**.37**	**.31**	**.37**	**.59**	**.22**	**.28**	**.54**	**.78**	**.74**	1.0								
11. Feed	**.22**	**.17**	**.20**	**.42**	**.19**	**.21**	**.49**	**.50**	**.43**	**.48**	1.0							
12. Help with incontinence	**.22**	**.31**	**.16**	**.37**	**.24**	**.40**	**.47**	**.56**	**.56**	**.58**	**.54**	1.0						
13. Give medicines	**.33**	**.31**	**.29**	**.40**	**.25**	**.26**	**.45**	**.50**	**.49**	**.56**	**.53**	**.45**	1.0					
14. Prepare food for special diets	**.17**	**.31**	**.38**	**.61**	**.10**	**.29**	**.34**	**.35**	**.32**	**.34**	**.50**	**.34**	**.44**	1.0				
16. Wound care	**.17**	**.21**	**.24**	**.32**	.02	**.21**	**.37**	**.45**	**.45**	**.42**	**.22**	**.42**	**.32**	**.32**	1.0			
17. Monitor blood pressure	**.29**	**.36**	**.20**	**.22**	**.33**	**.27**	**.37**	**.31**	**.33**	**.31**	**.31**	**.32**	**.47**	**.41**	**.21**	1.0		
18. Monitor blood sugar	**.29**	**.38**	**.20**	**.21**	**.23**	**.23**	**.31**	**.33**	**.26**	**.33**	**.39**	**.37**	**.45**	**.41**	**.22**	**.71**	1.0	
19. Operate special equipment	**.28**	**.23**	**.19**	**.23**	**.15**	**.24**	**.51**	**.43**	**.53**	**.41**	**.26**	**.36**	**.28**	**.13**	**.40**	**.31**	**.24**	1.0

Note. **Bolded** values are significant at $p<.05$.

Table S2.

Factors and Eigenvalues

Factor	Eigenvalue	Difference	Proportion	Cumulative
**Factor1**	**6.474**	5.247	0.841	0.841
**Factor2**	**1.227**	0.364	0.159	1.000
**Factor3**	0.863	0.383	0.112	1.112
**Factor4**	0.480	0.076	0.062	1.174
**Factor5**	0.404	0.209	0.052	1.227
**Factor6**	0.194	0.061	0.025	1.252
**Factor7**	0.133	0.064	0.017	1.269
**Factor8**	0.069	0.070	0.009	1.278
**Factor9**	-0.002	0.003	0.000	1.278
**Factor10**	-0.005	0.073	-0.001	1.277
**Factor11**	-0.078	0.057	-0.010	1.267
**Factor12**	-0.135	0.065	-0.018	1.250
**Factor13**	-0.200	0.035	-0.026	1.224
**Factor14**	-0.235	0.054	-0.031	1.193
**Factor15**	-0.289	0.041	-0.038	1.156
**Factor16**	-0.330	0.089	-0.043	1.113
**Factor17**	-0.419	0.032	-0.054	1.059
**Factor18**	-0.451	.	-0.059	1.000

Table S3.

Factor Loadings, Uniqueness, and Communalities for Informal Caregiving Activities

Variables	Factor 1	Factor 2	Uniqueness	Communality
Activities of Daily Living				
Getting in and out of beds and chairs	**0.811**	-0.059	0.387	0.613
Getting dressed	**0.872**	-0.059	0.288	0.712
Getting to and from the toilet	**0.942**	-0.141	0.226	0.774
Bathing or showering	**0.804**	0.057	0.304	0.696
Feeding	**0.461**	0.246	0.612	0.388
Instrumental Activities of Daily Living				
Transportation	0.216	**0.321**	0.781	0.219
Housework	**0.349**	0.136	0.812	0.188
Grocery Shopping	0.144	**0.451**	0.711	0.289
Preparing Meals	**0.563**	0.135	0.588	0.412
Managing Finances	-0.121	**0.498**	0.798	0.202
Arranging Outside Services	0.086	**0.408**	0.791	0.209
Medical & Nursing Tasks				
Help with incontinence	**0.549**	0.216	0.533	0.467
Giving medicines like pills, eye drops, or injections	**0.412**	0.399	0.506	0.494
Preparing special diets, administering tube feeding	0.271	**0.410**	0.647	0.353
Wound care	**0.526**	0.016	0.715	0.285
Monitoring blood pressure	0.019	**0.744**	0.432	0.568
Monitoring blood sugar	0.019	**0.727**	0.457	0.543
Helping with incontinence	**0.505**	0.059	0.712	0.288
Operating equipment like hospital beds, wheelchairs, oxygen tanks, nebulizers, or suctioning tubes	**0.412**	0.399	0.506	0.467

Note. Giving medicines like pills, eye drops, or injections loaded on both Factor 1 and Factor 2. When creating the composites, it was included in the scores for only Factor 1. Uniqueness = the portion of the variance in a particular variable that is not accounted for by the underlying, extracted factors.

Table S4.

Factor Rotation Matrix

	Factor1	Factor2
Factor1	0.951	0.745
Factor2	-0.308	0.668

Table S5.

Standardized Coefficients for Resilience and Social Support Resources Split into Subscales across Informal Caregiver Motivation Class

	Duty Motives	Affective Motives	Obligation Motives	Cultural Motives	Situational Motives
**Self-Esteem**	0.283^{a1}	0.018^{ab1}	-0.125^{b12}	0.177^{a1}	-0.129^{b12}
**Mastery**	0.230^{a1}	0.024^{a1}	0.057^{ab1}	0.073^{ab1}	-0.213^{b1}
**Optimism**	0.161^{a1}	0.041^{a1}	-0.237^{b2}	0.234^{a1}	-0.058^{ab12}
**Spirituality/Religion**	0.397^{a12}	-0.015^{bd1}	-0.319^{cd2}	0.363^{a1}	-0.055^{d12}
**Familism**	-0.028^{ab3}	0.066^{ab1}	-0.171^{a1}	0.189^{b1}	-0.058^{ab12}
**Social-Support Seeking**	-0.029^{a13}	0.005^{a1}	-0.155^{a1}	0.145^{a1}	0.053^{a2}

Note. When superscript letters in a particular row differ (i.e., one column is “a” and the other column is “b”), the mean standardized coefficients for those two comparisons differ significantly at the $p<.05$ level. When superscript numbers in a particular column differs (i.e., one row is “1” and the other is “2”), the mean standardized coefficients for those two comparisons differ significantly at the $p<.05$ level.

Table S6. *Multinomial Logistic Regression Predicting Class Membership from Demographics, Health Challenges of the Person Needing Care, and Informal Caregiver Resources*

Variables	Duty Motives			Affective Motives			Obligation Motives			Cultural Motives		
	RRR	SE	95th% CI	RRR	SE	95th % CI	RRR	SE	95th % CI	RRR	SE	95th % CI
CG Age	1.47***	0.10	1.30, 1.67	1.03	0.04	0.95, 1.11	1.16**	0.07	1.03, 1.29	0.93*	0.03	0.88, 0.99
CG Gender	0.56**	0.10	0.39, 0.80	1.39***	0.07	1.26, 1.53	1.74***	0.18	1.42, 2.14	1.41*	0.23	1.03, 1.94
CG AIAN	3.23***	0.40	2.54, 4.11	1.00	0.10	0.82, 1.22	1.66***	0.17	1.36, 2.04	4.88***	1.26	2.95, 8.08
CG Asian	8.86***	2.20	5.44, 14.42	1.98***	0.45	1.26, 3.10	0.24***	0.05	0.16, 0.36	12.80***	3.07	8.00, 20.49
CG Black	1.30	0.20	0.96, 1.77	1.50***	0.12	1.28, 1.74	0.89	0.15	0.64, 1.24	2.21***	0.28	1.72, 2.84
CG Other	1.58	0.92	0.51, 4.92	0.36***	0.13	0.18, 0.71	0.33*	0.18	0.11, 0.95	1.15	0.64	0.39, 3.44
CG Hispanic/ Latino	2.10***	0.27	1.63, 2.70	0.60***	0.02	0.56, 0.65	0.46***	0.07	0.34, 0.62	3.28***	0.12	3.05, 3.53
CG Education	1.51***	0.08	1.36, 1.68	1.09***	0.03	1.03, 1.14	0.99	0.03	0.94, 1.04	1.72***	0.06	1.61, 1.83
CG Income	1.16***	0.04	1.08, 1.24	1.06*	0.03	1.01, 1.12	1.30***	0.06	1.18, 1.43	1.21***	0.02	1.18, 1.24
CG Stopped Working	1.83***	0.25	1.40, 2.41	0.96	0.07	0.84, 1.09	1.97***	0.16	1.68, 2.31	1.40*	0.19	1.08, 1.81
CG Employed	1.71***	0.18	1.40, 2.10	0.95	0.04	0.87, 1.04	1.92***	0.09	1.74, 2.10	1.48***	0.12	1.26, 1.73
CG Partnered	0.56***	0.06	0.46, 0.68	1.23***	0.03	1.17, 1.30	1.14***	0.05	1.06, 1.24	1.26**	0.10	1.07, 1.49
CG Divorced	0.82	0.14	0.59, 1.13	0.87	0.12	0.66, 1.15	0.57	0.17	0.31, 1.03	0.80*	0.07	0.67, 0.95
CG Widowed	2.42**	0.73	1.35, 4.36	2.02***	0.22	1.64, 2.49	1.04	0.08	0.90, 1.20	2.49***	0.53	1.63, 3.78
CR Age	1.16	0.10	0.97, 1.38	0.89**	0.04	0.82, 0.97	1.10	0.11	0.91, 1.34	1.42***	0.09	1.26, 1.61
CR Gender	1.10	0.08	0.96, 1.26	1.44***	0.04	1.36, 1.53	1.34***	0.12	1.12, 1.61	1.92***	0.13	1.68, 2.20
CR Lives w/ Someone	2.99***	0.18	2.66, 3.36	1.12*	0.06	1.02, 1.24	1.47***	0.07	1.34, 1.62	2.15***	0.30	1.63, 2.83
CR Lives in Facility	1.16	0.27	0.73, 1.84	1.89***	0.18	1.57, 2.27	0.97	0.09	0.80, 1.17	1.08	0.12	0.86, 1.34
CR Lives w/ CG	0.71	0.17	0.45, 1.12	1.16	0.18	0.85, 1.56	1.74**	0.35	1.17, 2.59	0.47***	0.11	0.30, 0.73
CR Has Spouse	0.89	0.10	0.72, 1.11	1.63***	0.05	1.53, 1.73	1.13*	0.06	1.02, 1.25	1.83***	0.13	1.60, 2.10

Table continues on next page

Variables	Duty Motives			Affective Motives			Obligation Motives			Cultural Motives		
	RRR	SE	95th% CI	RRR	SE	95th % CI	RRR	SE	95th % CI	RRR	SE	95th % CI
CR is Spouse	9.39***	1.05	7.54, 11.70	2.73***	0.37	2.09, 3.56	7.69***	1.52	5.22, 11.32	0.56*	0.13	0.35, 0.89
CR is Parent	3.88***	0.36	3.24, 4.66	2.20***	0.11	1.99, 2.43	3.44***	0.31	2.87, 4.11	1.72***	0.19	1.39, 2.12
CR is Grandparent	3.12***	0.71	1.99, 4.88	3.41***	0.14	3.14, 3.70	2.33***	0.38	1.68, 3.22	1.98***	0.27	1.52, 2.58
CR is Older Relative	0.91	0.21	0.59, 1.42	2.79***	0.07	2.65, 2.93	2.48***	0.19	2.13, 2.89	1.80***	0.31	1.30, 2.51
CR is Child	2.50***	0.47	1.73, 3.61	1.67***	0.11	1.47, 1.90	9.70***	1.88	6.63, 14.18	1.73***	0.18	1.42, 2.12
CR is Sibling/ Cousin	7.23***	2.03	4.17, 12.53	2.42***	0.14	2.16, 2.71	8.26***	0.91	6.65, 10.26	2.13***	0.46	1.39, 3.26
Short-term physical	1.68*	0.34	1.12, 2.51	1.14***	0.03	1.09, 1.20	1.45***	0.04	1.37, 1.54	0.67**	0.09	0.52, 0.87
Long-term physical	4.79***	0.44	4.00, 5.72	1.94***	0.20	1.58, 2.37	3.23***	0.35	2.60, 4.00	1.56***	0.14	1.30, 1.87
Chronic	2.46***	0.23	2.05, 2.95	1.35***	0.09	1.19, 1.54	1.89***	0.15	1.62, 2.21	1.25*	0.11	1.04, 1.49
Mental health	1.41*	0.24	1.02, 1.96	1.44***	0.12	1.21, 1.70	2.37***	0.25	1.93, 2.91	1.75***	0.13	1.52, 2.03
Memory	1.69**	0.28	1.22, 2.34	1.15*	0.06	1.03, 1.28	1.42***	0.11	1.22, 1.65	0.96	0.16	0.69, 1.33
Behavioral	1.26	0.30	0.78, 2.02	0.70***	0.08	0.56, 0.87	0.82	0.14	0.59, 1.14	1.81*	0.50	1.05, 3.13
Intellectual/ Developmental	0.51***	0.10	0.35, 0.74	1.26	0.18	0.95, 1.67	1.63	0.45	0.95, 2.79	2.18***	0.52	1.36, 3.50
General aging concerns	2.00***	0.18	1.68, 2.37	1.54***	0.12	1.33, 1.80	2.18***	0.21	1.79, 2.64	1.26***	0.07	1.13, 1.39
Self-Esteem	1.44***	0.11	1.25, 1.67	1.14***	0.03	1.09, 1.19	1.22***	0.03	1.15, 1.29	1.14	0.09	0.98, 1.33
Mastery	1.39***	0.05	1.29, 1.49	1.23***	0.05	1.14, 1.33	1.58***	0.06	1.47, 1.70	1.10***	0.02	1.05, 1.14
Optimism	0.78***	0.04	0.71, 0.85	1.07**	0.02	1.02, 1.11	0.86***	0.03	0.80, 0.93	0.98	0.08	0.83, 1.15
Social-Support Seeking	0.67***	0.04	0.60, 0.76	1.34***	0.06	1.22, 1.47	0.95	0.06	0.84, 1.07	1.25**	0.10	1.06, 1.46
Familism	1.08	0.08	0.94, 1.24	0.74***	0.04	0.67, 0.82	0.99	0.05	0.90, 1.08	0.75***	0.04	0.68, 0.83
Spiritual/ Religious	1.62***	0.14	1.37, 1.93	0.83***	0.02	0.79, 0.86	0.69***	0.03	0.63, 0.75	1.46***	0.04	1.38, 1.55

*p<.05, **p<.01; ***p<.001; RRR = Relative Risk Ratio, SE = standard error, CI = confidence interval, CG = informal caregiver; CR = Care Recipient

Table S7. *Average Marginal Effects Associated with the Multinomial Logistic Regression Predicting Class Membership from Demographics, Health Challenges of the Person Needing Care, and Informal Caregiver Resources*

	**Duty Motives**			**Affective Motives**			**Obligation Motives**			**Cultural Motives**			**Situational Motives**		
**Variables**	**AME**	**SE**	**95% CI**	**AME**	**SE**	**95% CI**	**AME**	**SE**	**95% CI**	**AME**	**SE**	**95% CI**	**AME**	**SE**	**95% CI**
CG Age	0.024	0.02	-0.01, 0.06	-0.011	0.02	-0.05, 0.02	0.012	0.01	-0.01, 0.04	-0.014	0.01	-0.04, 0.01	-0.011	0.01	-0.04, 0.02
CG Gender	**-0.066**	0.05	-0.16, 0.03	0.040	0.04	-0.03, 0.11	**0.052**	0.05	-0.05, 0.15	0.019	0.01	0, 0.04	-0.045	0.03	-0.10, 0.01
CG AIAN	**0.051**	0.05	-0.04, 0.14	**-0.140**	0.08	-0.31, 0.03	0.010	0.04	-0.07, 0.09	**0.156**	0.09	-0.03, 0.34	**-0.077**	0.08	-0.23, 0.08
CG Asian	**0.124**	0.10	-0.07, 0.32	**-0.065**	0.12	-0.31, 0.18	**-0.177**	0.15	-0.48, 0.12	**0.251**	0.13	-0.01, 0.51	**-0.134**	0.10	-0.33, 0.06
CG Black	-0.003	0.01	-0.02, 0.02	**0.053**	0.03	0, 0.11	**-0.055**	0.05	-0.15, 0.04	**0.057**	0.04	-0.02, 0.13	**-0.052**	0.03	-0.11, 0.01
CG Other	**0.085**	0.09	-0.09, 0.25	**-0.163**	0.07	-0.31, -0.02	**-0.082**	0.09	-0.25, 0.09	**0.058**	0.09	-0.12, 0.23	**0.103**	0.10	-0.10, 0.30
CG Hispanic/ Latino	**0.062**	0.06	-0.05, 0.17	**-0.141**	0.08	-0.30, 0.02	**-0.090**	0.09	-0.26, 0.08	**0.156**	0.11	-0.05, 0.36	0.013	0.04	-0.07, 0.09
CG Education	0.019	0.02	-0.01, 0.05	-0.014	0.02	-0.05, 0.02	-0.020	0.02	-0.05, 0.02	0.039	0.03	-0.02, 0.10	-0.024	0.02	-0.06, 0.02
CG Income	0.002	0.00	-0.01, 0.01	-0.016	0.01	-0.04, 0.01	0.025	0.01	0, 0.05	0.009	0.01	-0.01, 0.03	-0.020	0.02	-0.05, 0.01
CG Stopped Working	0.028	0.03	-0.03, 0.08	**-0.084**	0.04	-0.17, 0	**0.077**	0.05	-0.01, 0.17	0.015	0.01	-0.01, 0.04	-0.035	0.04	-0.12, 0.05
CG Employed	0.023	0.02	-0.02, 0.06	**-0.084**	0.04	-0.17, 0	**0.075**	0.04	0, 0.15	0.020	0.02	-0.01, 0.05	-0.034	0.04	-0.11, 0.04
CG Partnered	**-0.050**	0.04	-0.12, 0.02	0.042	0.02	0, 0.09	0.009	0.01	-0.02, 0.04	0.018	0.02	-0.01, 0.05	-0.019	0.01	-0.03, 0
CG Divorced	0.001	0.01	-0.03, 0.03	0.020	0.03	-0.04, 0.08	**-0.053**	0.05	-0.16, 0.05	-0.005	0.02	-0.03, 0.02	0.038	0.05	-0.06, 0.13
CG Widowed	0.028	0.04	-0.05, 0.11	**0.079**	0.04	0.01, 0.15	**-0.063**	0.05	-0.16, 0.03	0.041	0.03	-0.03, 0.11	**-0.085**	0.05	-0.18, 0.01
CR Age	0.007	0.01	-0.01, 0.02	-0.049	0.02	-0.08, -0.02	0.013	0.01	-0.01, 0.04	0.033	0.02	-0.02, 0.08	-0.004	0.01	-0.02, 0.01
CR Gender	-0.016	0.01	-0.04, 0.01	0.032	0.02	-0.01, 0.08	0.004	0.02	-0.03, 0.04	0.037	0.03	-0.02, 0.09	**-0.057**	0.03	-0.12, 0.01
CR Lives w/ Someone	**0.066**	0.05	-0.03, 0.16	**-0.067**	0.04	-0.15, 0.01	0.011	0.01	-0.01, 0.03	0.045	0.03	-0.01, 0.10	**-0.055**	0.04	-0.14, 0.03
CR Lives in Facility	-0.009	0.02	-0.05, 0.03	**0.138**	0.01	0.12, 0.16	**-0.050**	0.03	-0.11, 0.01	-0.022	0.02	-0.06, 0.02	**-0.057**	0.04	-0.13, 0.02
CR Lives w/ CG	-0.025	0.03	-0.08, 0.03	0.020	0.04	-0.05, 0.09	**0.082**	0.05	-0.01, 0.18	**-0.061**	0.04	-0.15, 0.03	-0.016	0.03	-0.07, 0.04
CR Has Spouse	-0.029	0.02	-0.07, 0.01	**0.075**	0.03	0.02, 0.13	-0.025	0.02	-0.07, 0.02	0.034	0.03	-0.03, 0.10	**-0.056**	0.03	-0.11, 0

Table Continues on Next Page

Variables	Duty Motives			Affective Motives			Obligation Motives			Cultural Motives			Situational Motives		
	AME	SE	95% CI	AME	SE	95% CI	AME	SE	95% CI	AME	SE	95% CI	AME	SE	95% CI
CR is Spouse	**0.116**	0.09	-0.06, 0.29	-0.035	0.09	-0.21, 0.14	**0.172**	0.08	0.02, 0.32	**-0.099**	0.09	-0.28, 0.08	**-0.153**	0.11	-0.37, 0.07
CR is Parent	0.046	0.04	-0.03, 0.12	0.027	0.06	-0.08, 0.14	**0.078**	0.06	-0.03, 0.19	-0.017	0.03	-0.07, 0.03	**-0.133**	0.07	-0.27, 0.01
CR is Grandparent	0.020	0.02	-0.01, 0.06	**0.145**	0.05	0.04, 0.25	-0.010	0.03	-0.07, 0.05	-0.018	0.03	-0.08, 0.04	**-0.138**	0.08	-0.30, 0.02
CR is Older Relative	-0.045	0.04	-0.12, 0.03	**0.131**	0.05	0.03, 0.23	0.031	0.05	-0.07, 0.13	-0.006	0.03	-0.07, 0.05	**-0.111**	0.07	-0.25, 0.03
CR is Child	-0.008	0.03	-0.06, 0.04	**-0.136**	0.09	-0.30, 0.03	**0.305**	0.07	0.16, 0.45	-0.029	0.03	-0.09, 0.04	**-0.132**	0.10	-0.33, 0.07
CR is Sibling/ Cousin	**0.074**	0.07	-0.07, 0.22	**-0.085**	0.09	-0.26, 0.09	**0.195**	0.09	0.02, 0.37	-0.033	0.04	-0.11, 0.05	**-0.150**	0.12	-0.38, 0.08
Short-term physical	0.033	0.02	0, 0.07	0.002	0.03	-0.05, 0.06	0.036	0.02	-0.01, 0.08	-0.048	0.03	-0.11, 0.01	-0.023	0.01	-0.05, 0
Long-term physical	**0.073**	0.05	-0.03, 0.18	-0.007	0.06	-0.13, 0.11	**0.078**	0.05	-0.03, 0.18	-0.026	0.02	-0.07, 0.02	**-0.118**	0.09	-0.29, 0.05
Chronic	0.043	0.03	-0.02, 0.11	-0.013	0.03	-0.08, 0.05	0.046	0.03	0, 0.10	-0.014	0.01	-0.04, 0.01	**-0.063**	0.05	-0.16, 0.04
Mental health	-0.007	0.01	-0.02, 0.01	-0.016	0.04	-0.09, 0.05	**0.078**	0.04	0, 0.16	0.019	0.02	-0.01, 0.05	**-0.073**	0.04	-0.16, 0.01
Memory	0.029	0.03	-0.03, 0.08	-0.008	0.02	-0.05, 0.03	0.029	0.03	-0.02, 0.08	-0.020	0.02	-0.06, 0.02	-0.029	0.03	-0.09, 0.03
Behavioral	0.019	0.03	-0.04, 0.08	**-0.095**	0.04	-0.18, -0.01	-0.016	0.03	-0.07, 0.04	**0.078**	0.06	-0.03, 0.19	0.014	0.02	-0.02, 0.05
Intellectual/ Developmental	**-0.052**	0.04	-0.14, 0.04	-0.011	0.03	-0.07, 0.04	0.042	0.04	-0.03, 0.12	**0.068**	0.05	-0.02, 0.16	-0.048	0.03	-0.11, 0.02
General aging concerns	0.021	0.02	-0.01, 0.06	0.012	0.03	-0.05, 0.07	**0.058**	0.04	-0.02, 0.14	-0.017	0.02	-0.05, 0.02	**-0.075**	0.04	-0.15, 0
Self-Esteem	0.017	0.01	-0.01, 0.04	0.001	0.01	-0.02, 0.02	0.009	0.01	-0.01, 0.03	-0.001	0.01	-0.01, 0.01	-0.026	0.02	-0.06, 0.01
Mastery	0.009	0.01	-0.01, 0.02	0.002	0.01	-0.03, 0.03	0.039	0.02	0, 0.08	-0.010	0.01	-0.03, 0.01	-0.039	0.02	-0.08, 0
Optimism	-0.016	0.01	-0.04, 0	0.032	0.01	0.01, 0.05	-0.019	0.01	-0.05, 0.01	0.001	0.00	-0.01, 0.01	0.003	0.01	-0.02, 0.03
Social-Support Seeking	-0.036	0.03	-0.1, 0.02	**0.069**	0.02	0.03, 0.11	-0.024	0.02	-0.07, 0.02	0.014	0.02	-0.02, 0.05	-0.022	0.02	-0.05, 0.01
Familism	0.017	0.01	-0.01, 0.04	**-0.054**	0.01	-0.08, -0.03	0.021	0.02	-0.01, 0.06	-0.014	0.01	-0.04, 0.01	0.031	0.02	-0.01, 0.07
Spiritual/ Religious	0.038	0.03	-0.02, 0.10	-0.044	0.03	-0.10, 0.01	-0.047	0.03	-0.11, 0.02	0.040	0.03	-0.02, 0.10	0.013	0.02	-0.02, 0.04

AME = Average Marginal Effect, SE = standard error, CI = confidence interval, CG = informal caregiver; CR = Care Recipient; Note. All bolded AMEs are either ≥ 5 percentage points or ≤ -5 percentage points.

Table S8. Unadjusted and Adjusted Predictors of Acute Emotional Distress Across Informal Caregiving Motivation Profiles

Variables	Unadjusted Acute Emotional Distress				Adjusted Acute Emotional Distress			
	β	SE	95th % CI		β	SE	95th % CI	
			Low	High			Low	High
Duty Motives	0.028***	0.002	0.025	0.032	0.045	0.035	-0.024	0.115
Affective Motives	0.070***	0.002	0.067	0.074	0.051**	0.018	0.016	0.085
Obligation Motives	0.171***	0.002	0.167	0.175	0.168***	0.024	0.121	0.215
Culture Motives	0.252***	0.002	0.248	0.255	0.155***	0.026	0.105	0.206
Resilience	-0.149***	0.016	-0.180	-0.118	-0.137***	0.029	-0.193	-0.081
Social Support	-0.166***	0.026	-0.217	-0.115	-0.169***	0.042	-0.251	-0.087
Caregiving Hours/Week	-0.047***	0.000	-0.048	-0.047	-0.044*	0.021	-0.086	-0.002
Caregiving Years	0.062***	0.001	0.061	0.064	0.010	0.015	-0.021	0.040
Intensive Tasks	0.051***	0.001	0.049	0.053	-0.004	0.009	-0.020	0.013
Coordination & Management Tasks	0.094***	0.000	0.094	0.094	0.123***	0.011	0.101	0.145
Duty Motives by								
Intensive Tasks	0.046***	0.001	0.044	0.047	0.184***	0.003	0.177	0.191
Coordination & Management Tasks	-0.221***	0.000	-0.221	-0.221	-0.291***	0.021	-0.333	-0.249
Caregiving Hours/Week	0.143***	0.000	0.143	0.144	0.035	0.025	-0.013	0.083
Caregiving Years	-0.153***	0.001	-0.154	-0.151	-0.121***	0.026	-0.172	-0.070
Affective Motives by								
Intensive Tasks	-0.007***	0.001	-0.009	-0.005	0.106***	0.008	0.091	0.122
Coordination & Management Tasks	-0.057***	0.000	-0.057	-0.057	-0.082***	0.014	-0.109	-0.055
Caregiving Hours/Week	0.097***	0.000	0.097	0.097	0.101***	0.003	0.095	0.107
Caregiving Years	-0.020***	0.001	-0.021	-0.018	0.030	0.019	-0.007	0.067
Obligation Motives by								
Intensive Tasks	0.109***	0.001	0.107	0.111	0.140***	0.010	0.119	0.160
Coordination & Management Tasks	-0.127***	0.000	-0.127	-0.127	-0.169***	0.013	-0.194	-0.144
Caregiving Hours/Week	0.186***	0.000	0.186	0.186	0.159***	0.007	0.145	0.174
Caregiving Years	-0.039***	0.001	-0.040	-0.038	0.073*	0.029	0.016	0.129
Culture Motives by								
Intensive Tasks	0.108***	0.001	0.107	0.110	0.026***	0.006	0.014	0.038
Coordination & Management Tasks	0.036***	0.000	0.035	0.036	0.055**	0.017	0.021	0.089
Caregiving Hours/Week	-0.029***	0.000	-0.030	-0.028	0.043***	0.007	0.028	0.058
Caregiving Years	-0.131	0.001	-0.133	-0.130	-0.085***	0.013	-0.112	-0.059

Table continues on next page

Variables	Unadjusted Acute Emotional Distress				Adjusted Acute Emotional Distress			
	β	SE	95th % CI		β	SE	95th % CI	
			Low	High			Low	High
Social Support by								
Duty Motives	-0.017	0.026	-0.067	0.034	0.057	0.042	-0.025	0.139
Affective Motives	0.010	0.026	-0.041	0.061	0.025	0.039	-0.050	0.101
Obligation Motives	0.011	0.026	-0.040	0.062	-0.004	0.043	-0.088	0.080
Culture Motives	0.151***	0.026	0.100	0.202	0.114*	0.045	0.025	0.202
Resilience x								
Duty Motives	-0.026***	0.015	-0.056	0.004	-0.139***	0.026	-0.191	-0.088
Affective Motives	-0.180***	0.016	-0.211	-0.149	-0.178***	0.028	-0.233	-0.123
Obligation Motives	-0.076***	0.015	-0.106	-0.046	-0.077***	0.020	-0.116	-0.038
Culture Motives	-0.266***	0.016	-0.297	-0.236	-0.216***	0.032	-0.279	-0.153
CG Gender					-0.079	0.080	-0.235	0.078
CG Age					-0.188***	0.019	-0.226	-0.151
CG Education					0.039*	0.016	0.007	0.070
CG Income					0.040	0.023	-0.005	0.085
CG Was Employed but Stopped					0.378***	0.066	0.250	0.507
CG is Employed					-0.435***	0.083	-0.598	-0.272
CG has a Partner					0.044*	0.016	0.013	0.075
CG is Divorced					0.101	0.074	-0.044	0.245
CG is Widowed					0.506***	0.142	0.227	0.784
CR Gender					0.007	0.065	-0.119	0.134
CR Age					-0.012	0.031	-0.073	0.050
CR Lives w/Someone Else					-0.063	0.066	-0.192	0.066
CR Lives in a Facility					0.037	0.096	-0.150	0.225
CR Lives with CG					-0.100	0.093	-0.283	0.083
CR Has a Spouse					-0.054	0.042	-0.137	0.030
CR is Immediate Family					0.122	0.078	-0.030	0.274
CR is Extended Family					0.094***	0.028	0.039	0.150
Total CR Health Challenges					0.034	0.023	-0.010	0.079
R^2 Total	0.175				0.356			

Note. Standardized regression coefficients (β), standard errors (SE), and 95% confidence intervals (CI) are presented. Both models include main effects of informal caregiving motivations, stressors (e.g., hours, years, and task types), and resources (resilience, social support), as well as interaction terms capturing moderation by each motivation class. Adjusted models additionally control for informal caregiver and care recipient demographic covariates.

*p < .05, **p < .01, **p < .001

Table S9. Unadjusted and Adjusted Predictors of Chronic Burnout Across Informal Caregiving Motivation Profiles

Variables	Unadjusted Chronic Burnout				Adjusted Chronic Burnout			
	β	SE	95th % CI		β	SE	95th % CI	
			Low	High			Low	High
Duty Motives	0.055***	0.004	0.047	0.063	-0.017	0.025	-0.066	0.032
Affective Motives	-0.017***	0.005	-0.026	-0.008	-0.063***	0.010	-0.082	-0.044
Obligation Motives	0.145***	0.005	0.136	0.154	0.101***	0.013	0.076	0.127
Culture Motives	0.141***	0.004	0.132	0.150	0.066**	0.022	0.022	0.109
Resilience	-0.223***	0.038	-0.297	-0.149	-0.204***	0.043	-0.289	-0.120
Social Support	-0.229***	0.063	-0.352	-0.106	-0.242***	0.069	-0.377	-0.107
Caregiving Hours/Week	-0.052***	0.001	-0.054	-0.051	-0.042**	0.016	-0.073	-0.011
Caregiving Years	0.068***	0.001	0.065	0.071	0.032***	0.005	0.022	0.042
Intensive Tasks	0.002	0.002	-0.002	0.006	-0.001	0.011	-0.023	0.020
Coordination & Management Tasks	0.261***	0.000	0.261	0.262	0.247***	0.013	0.222	0.273
Duty Motives by								
Intensive Tasks	0.220***	0.002	0.216	0.224	0.225***	0.007	0.212	0.238
Coordination & Management Tasks	-0.300***	0.000	-0.301	-0.300	-0.344***	0.023	-0.390	-0.298
Caregiving Hours/Week	-0.070***	0.001	-0.072	-0.069	-0.053**	0.021	-0.093	-0.013
Caregiving Years	-0.156***	0.002	-0.160	-0.152	-0.093***	0.021	-0.134	-0.051
Affective Motives by								
Intensive Tasks	0.140***	0.003	0.135	0.145	0.146***	0.007	0.133	0.158
Coordination & Management Tasks	-0.210***	0.000	-0.211	-0.210	-0.227***	0.010	-0.248	-0.207
Caregiving Hours/Week	0.131***	0.000	0.131	0.131	0.136***	0.003	0.131	0.142
Caregiving Years	-0.062***	0.002	-0.066	-0.059	-0.048***	0.014	-0.075	-0.022
Obligation Motives by								
Intensive Tasks	0.081***	0.002	0.076	0.085	0.099***	0.010	0.079	0.120
Coordination & Management Tasks	-0.184***	0.000	-0.185	-0.184	-0.232***	0.007	-0.245	-0.219
Caregiving Hours/Week	0.049***	0.000	0.048	0.049	0.041***	0.006	0.029	0.053
Caregiving Years	-0.054***	0.001	-0.057	-0.051	-0.007	0.022	-0.050	0.035
Culture Motives by								
Intensive Tasks	0.142***	0.002	0.138	0.146	0.164***	0.014	0.136	0.191
Coordination & Management Tasks	-0.085***	0.001	-0.086	-0.084	-0.129***	0.014	-0.156	-0.101
Caregiving Hours/Week	0.027***	0.001	0.025	0.029	0.048***	0.007	0.034	0.062
Caregiving Years	-0.104***	0.002	-0.108	-0.100	-0.059***	0.008	-0.074	-0.043

Table continues on next page

Variables	Unadjusted Chronic Burnout				Adjusted Chronic Burnout			
	β	SE	95th % CI		β	SE	95th % CI	
			Low	High			Low	High
Social Support by								
Duty Motives	0.109	0.062	-0.012	0.231	0.143*	0.063	0.019	0.267
Affective Motives	0.034	0.063	-0.088	0.157	0.055	0.069	-0.079	0.189
Obligation Motives	-0.008	0.062	-0.130	0.114	0.025	0.069	-0.110	0.160
Culture Motives	0.044	0.063	-0.078	0.167	0.065	0.064	-0.061	0.191
Resilience x								
Duty Motives	-0.092*	0.037	-0.164	-0.020	-0.108**	0.038	-0.182	-0.035
Affective Motives	-0.101***	0.038	-0.176	-0.027	-0.109*	0.044	-0.194	-0.023
Obligation Motives	-0.119***	0.037	-0.192	-0.047	-0.107**	0.035	-0.176	-0.038
Culture Motives	-0.139***	0.037	-0.212	-0.066	-0.122***	0.036	-0.192	-0.052
CG Gender					-0.046	0.071	-0.186	0.094
CG Age					-0.106***	0.024	-0.152	-0.059
CG Education					0.030	0.016	-0.002	0.062
CG Income					0.029	0.030	-0.030	0.088
CG Was Employed but Stopped					0.070	0.048	-0.024	0.165
CG is Employed					0.152*	0.065	0.026	0.279
CG has a Partner					0.007	0.036	-0.063	0.077
CG is Divorced					0.041	0.094	-0.144	0.226
CG is Widowed					0.239	0.127	-0.009	0.488
CR Gender					-0.049	0.050	-0.147	0.048
CR Age					-0.061	0.031	-0.122	-0.001
CR Lives w/Someone Else					0.021	0.073	-0.121	0.164
CR Lives in a Facility					0.302***	0.091	0.123	0.481
CR Lives with CG					-0.016	0.050	-0.114	0.082
CR Has a Spouse					-0.022	0.026	-0.072	0.029
CR is Immediate Family					0.196***	0.051	0.096	0.296
CR is Extended Family					0.157	0.087	-0.014	0.329
Total CR Health Challenges					0.101***	0.018	0.065	0.136
R^2 Total	0.257				0.292			

Note. Standardized regression coefficients (β), standard errors (SE), and 95% confidence intervals (CI) are presented. Both models include main effects of informal caregiving motivations, stressors (e.g., hours, years, and task types), and resources (resilience, social support), as well as interaction terms capturing moderation by each motivation class. Adjusted models additionally control for informal caregiver and care recipient demographic covariates. *p < .05, **p < .01, **p < .001
